# Density dependence can obscure nonlethal effects of disturbance on life history of medium-sized cetaceans

**DOI:** 10.1371/journal.pone.0252677

**Published:** 2021-06-03

**Authors:** Vincent Hin, John Harwood, André M. de Roos

**Affiliations:** 1 Department of Theoretical and Computational Ecology, Institute for Biodiversity and Ecosystem Dynamics, University of Amsterdam, Amsterdam, The Netherlands; 2 Centre for Research into Ecological and Environmental Modelling, University of St Andrews, St Andrews, United Kingdom; MARE – Marine and Environmental Sciences Centre, PORTUGAL

## Abstract

Nonlethal disturbance of animals can cause behavioral and physiological changes that affect individual health status and vital rates, with potential consequences at the population level. Predicting these population effects remains a major challenge in ecology and conservation. Monitoring fitness-related traits may improve detection of upcoming population changes, but the extent to which individual traits are reliable indicators of disturbance exposure is not well understood, especially for populations regulated by density dependence. Here we study how density dependence affects a population’s response to disturbance and modifies the disturbance effects on individual health and vital rates. We extend an energy budget model for a medium-sized cetacean (the long-finned pilot whale *Globicephala melas*) to an individual-based population model in which whales feed on a self-replenishing prey base and disturbance leads to cessation of feeding. In this coupled predator-prey system, the whale population is regulated through prey depletion and the onset of yearly repeating disturbances on the whale population at carrying capacity decreased population density and increased prey availability due to reduced top-down control. In populations faced with multiple days of continuous disturbance each year, female whales that were lactating their first calf experienced increased mortality due to depletion of energy stores. However, increased prey availability led to compensatory effects and resulted in a subsequent improvement of mean female body condition, mean age at first reproduction and higher age-specific reproductive output. These results indicate that prey-mediated density dependence can mask negative effects of disturbance on fitness-related traits and vital rates, a result with implications for the monitoring and management of marine mammal populations.

## Introduction

A major challenge in ecology and conservation is to understand and predict how populations respond to natural and human-induced changes in their environment. In the extreme case, environmental changes can trigger a regime shift and lead to a sudden population collapse [1–3]. More often the response to exploitation, pollution or climate change [4] is a gradual decline in population density. Stressors that drive population change can either have a direct, lethal effect or act in an indirect, nonlethal way by altering the physiology and behavior of animals [5, 6]. If these nonlethal effects change individual vital rates, such as the probability of survival or giving birth, they can alter population growth rate or density [7]. Assessing the population consequences of exposure to nonlethal stressors is required under many regulatory frameworks (e.g., European Habitats Directive 92/43/EEC, United States Marine Mammal Protection Act, 16 U.S.C. §§ 1361 et seq., and Endangered Species Act, 16 U.S.C. §1531 et seq.), but it is often challenging to monitor populations at the appropriate scale in order to detect and avert a population decline in a timely way [8, 9]. Monitoring fitness-related traits, such as body size or body condition, as well as population density can improve the detectability of an upcoming population collapse [10–12]. However, the use of fitness-related traits as an early warning signal for population decline relies on the assumption that shifts in the trait distribution are the result of changes in environmental conditions [13].

Marine mammals are exposed to a wide range of anthropogenic, nonlethal disturbances, such as noise from military sonar, pile driving or ship traffic, which may affect their physiology and behavior [14–16]. In addition, except for land-breeding species, density changes are hard to monitor with sufficient accuracy due to the elusive appearance of some species and the inaccessibility of the marine environment. Finally, many populations are recovering from historical exploitation and may be approaching levels at which density dependence may be an important regulatory process [17, 18]. The Population Consequences of Disturbance (PCoD) conceptual framework was developed to assess the effects of non-lethal disturbances on marine mammal populations [5, 6]. It links behavioral and physiological responses to disturbance, to the fitness-related traits that influence vital rates and population dynamics [7]. Bio-energetic models have been used to implement the PCoD framework for a number of marine mammal species [19–21]. For example, Hin et al. [21] developed an energy budget model of the long-finned pilot whale (*Globicephala melas*) to study the effects of disturbances that led to cessation of foraging, which resulted in the depletion of an individual’s energy stores. This model revealed that short disturbances decreased survival among calves born to young females, while longer disturbances also reduced survival of calves born to older females and degraded female survival itself. This led to an overall decrease in expected lifetime reproductive output (*R*_*0*_: the expected number of female calves born to a female during her life) with increasing disturbance. Hin et al. [21] and most other implementations of the PCoD framework model density-independent population dynamics, in which disturbance reduces survival and reproduction through negative effects on fitness-related traits and the population grows or declines exponentially depending on whether *R*_*0*_ is greater or smaller than 1 (but see [22, 23]).

Marine mammals generally have low predation rates and high adult survival. In similar populations of terrestrial, large-sized mammals, population regulation is thought to act primarily through resource / prey limitation [24–26]. In coupled predator–prey systems, top-down control of prey will suppress vital rates until the predator population attains a stationary state (carrying capacity), in which the expected lifetime reproductive output of a female equals 1 (*R*_*0*_ = 1). Disturbing a population at carrying capacity may have immediate effects on individual condition, vital rates and population density, but the subsequent release of density-dependent processes might compensate for these effects. Currently, it is not well understood how populations regulated by density dependence through prey depletion respond to disturbance when at carrying capacity, nor whether fitness-related traits are reliable indicators of the level of disturbance in such populations.

Here we use the energy budget model of Hin et al. [21] to explore how nonlethal disturbance in the form of lost foraging days affect individual body condition, vital rates and population dynamics for a marine mammal population that is regulated by density dependence acting through depletion of its prey. Although specifically tailored for pilot whales, the model of Hin et al. [21] provides a realistic representation of how the life history of a medium-sized cetacean arises from the dynamics of energy acquisition (through prey foraging) and energy expenditure (on metabolism, growth, lactation and gestation). We extend this model to a population context by modeling multiple individuals (females and males) and accounting for the feedback between the whale population and a self-replenishing prey base, which is depleted through foraging by the whale population. We subsequently use this model to show that disturbance decreases whale population density at carrying capacity and increases prey availability. The increase in prey availability leads to compensatory changes in body condition, age at first reproduction and reproductive output of female whales.

## Model

Pilot whales are highly social oceanic delphinids (Fam: *Delphinidae*) that have a matrilineal school structure and can form large aggregations (> 100 individuals, [27]). They perform deep dives to feed on cephalopods and several fish species [28–30]. Although specifically tailored for pilot whales, the model can represent any medium-sized cetacean for which long-distance migration is absent and feeding and reproduction occur continuously throughout the year.

The individual-based population model followed multiple generations of male and female pilot whales and their prey base. Each modeled whale required energy to meet the costs of metabolism and growth in body size. Female whales required additional energy for gestation (metabolic and growth costs of their fetus) and lactation. Energy was derived from prey feeding and all whales were assumed to feed from the same prey base. Each whale could also use its stored energy reserves, which increased if total energy acquisition exceeded total energy expenditure and decreased if the opposite was true. Ultimately, the ability of whales to grow, survive and reproduce (*i*.*e*. give birth to and wean a calf) was determined by prey density *R(t)*. Continuous-time dynamics of *R(t)* were described by the ordinary differential equation (ODE):

$$\frac{dR(t)}{dt}=K\left(1.0-A\cos(\frac{2\pi t}{365})\right)-\delta R(t)-V^{-1}\sum_{i}I_{R}(R,a_{i},S_{i},F_{i},W_{i})$$ (1)

Prey density was increased via a productivity term that could vary seasonally according to a cosine function with a period of 365 days and a relative amplitude *A* (0–1) that is proportional to, but independent of, the mean annual productivity *K*. Seasonal variation in prey productivity may be caused by seasonal prey migration, changes in ocean productivity, or the short, semelparous life histories of most cephalopod species that are the main prey of pilot whales [28]. Prey density is decreased through a first-order decay term (*δR*(*t*)) that accounted for prey mortality from sources other than pilot whale foraging. The last term in Eq (1) represents the decrease in prey density through total foraging of all pilot whale individuals. The prey feeding rate of an individual whale with index *i* (*I*_*R*_(*R*, *a*_*i*_, *S*_*i*_, *F*_*i*_, *W*_*i*_)) was a function of prey density *R*, whale age *a*, and body mass measures *S*, *F* and *W* (defined below). The volume scalar *V* converted the volumetric density of whales to the volume of prey.

The coupled dynamics between whales and their prey created competition between individual whales for available prey. Because prey density limited the energy available for growth, reproduction and survival, whale demographic rates were indirectly dependent on whale population density, through the depletion of prey. The resulting relationship between demographic rates and population density was thus an emergent property of the model rather than a formal assumption, as is the case for most models of density dependence in marine mammal populations [31].

### Energy budget model

We used the energy budget model of Hin et al. [21], which was based on a model for ungulates initially formulated by De Roos et al. [32]. Model parameters and equations are listed in Tables 1 and 2 in S1 File together with a detailed description of their derivation. An example of the energetics and survival throughout the life of a female pilot whale (and her calves) is shown in Fig 4 in S2 File. Whales were characterized by age (*a* in days) and biomass compartments of structure (mass *S* in kg and length *l* in cm) and energy reserves (mass *F* in kg). Following Kooijman [33], structure and reserves were distinguished primarily by their function: structural mass could not be catabolized to fuel energetic needs, while reserve mass functioned as a buffer between incoming and outgoing energy. Reserve and structural mass therefore did not translate one-to-one to a specific biochemical compound; structural mass mainly represented bones and organs, while reserves are represented primarily by stored lipids, and to a lesser extent proteins and carbohydrates [33, 34]. Total mass *W* equaled the sum of structural and reserve mass (*W* = *S* + *F*) and included the structural mass of the fetus for pregnant females. As individuals grew in structural size, they increased their capacity to store energy reserves. ‘Body condition’, defined as the ratio between reserve mass and total mass (*i*.*e*. *F / W*) was used as a standardized measure of individual health, which allowed the health status of differently sized individuals to be compared. We used this measure of body condition instead of the alternative formulation of *W* / *S* [33], because it is easier to relate to the data on lipid mass of pilot whales [34] that were used to parameterize our model.

**Fig 1:**
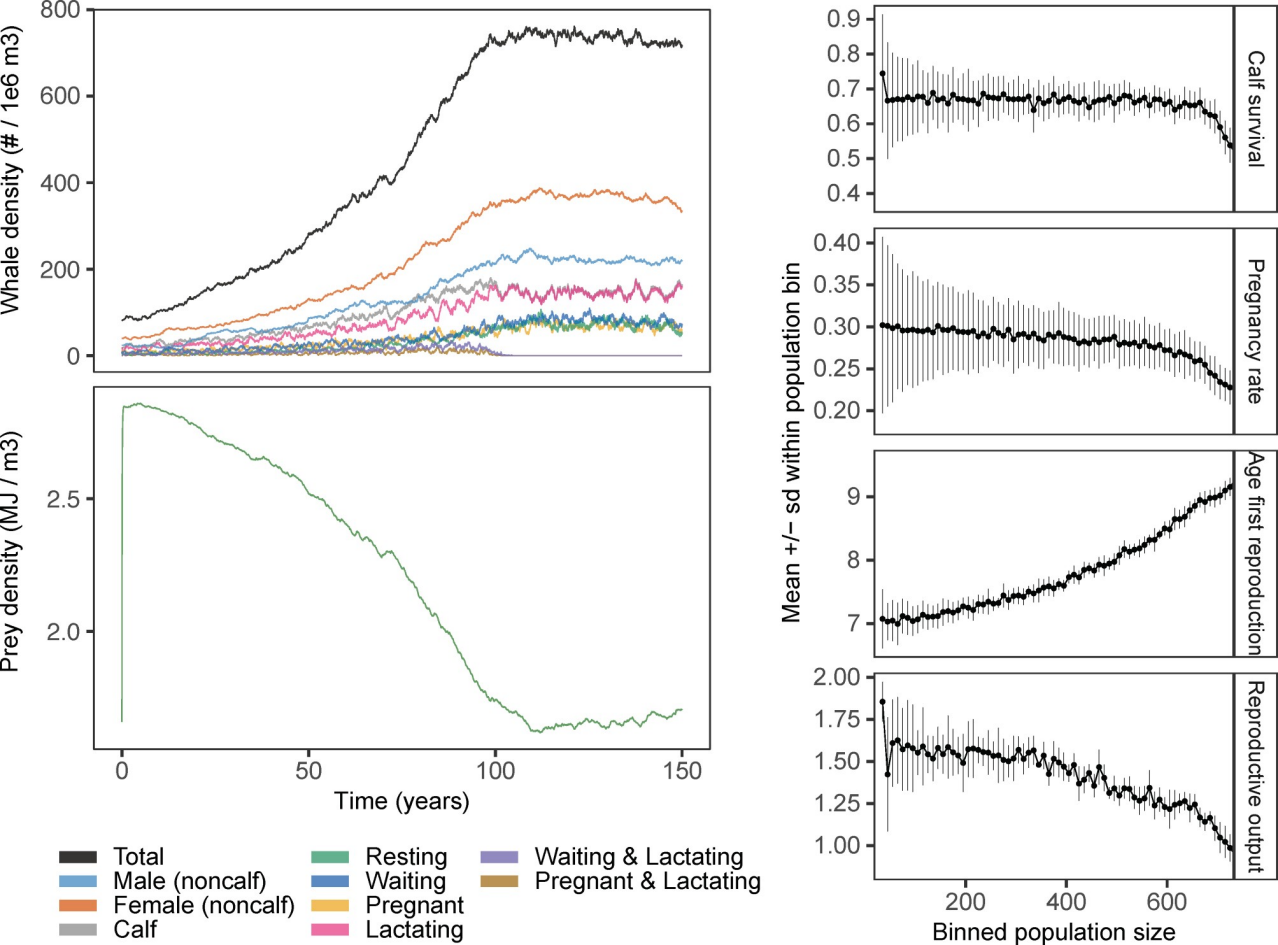
Density-dependent population dynamics. Changes in whale and prey densities and whale vital rates associated with the approach of the whale population to a stationary state. Left panels show the outcome of a single simulation with whale densities colored according to sex and reproductive status (for females only). Right panels show the mean +/- standard deviation of calf survival, pregnancy rate (ratio of pregnant to mature (i.e. age > 8 yrs.) females), female age at first reproduction (yrs.) and female lifetime reproductive output as a function of binned population size (80 bins of size 10 ranging from 0 to 800), derived from 100 replicate simulations. All replicate simulations were initialized with a unique stationary state at a mean annual prey productivity of *K* = 0.09 MJ m^-3^ day^-1^. Subsequently, *K* was increased to 0.15 at time zero for each simulation. All other parameters at default values (Table 1 in S1 File).

We assumed that growth in structural length is non-plastic and demand-driven, *i*.*e*. the growth rate and asymptotic length do not vary with the amount of assimilated energy. Instead, structural growth imposed a demand on energy intake. This contrasts with the supply-driven structural growth of other energy budget models [33, 35], which assume that asymptotic length depends on the rate of energy intake. We concluded that demand-driven growth [32] was more appropriate for this species, because the sex-specific variation in asymptotic length of pilot whales is small and growth patterns in relations to prey availability are uncertain [36]. Consequently, we use a fixed Von Bertalanffy structural length-age relationship: *l*(*a*) = *l*_∞_−(*l*_∞_−*l*_*b*_)*e*^−*ka*^. Data from Bloch et al. [36] were used to fit the asymptotic length *l*_∞_ and the Von Bertalanffy growth constant *k* separately for males and females (Fig 1 in S2 File). For *G*. *melas* in the North-East Atlantic, length at birth (*l*_*b*_) varied between 163 cm and 191 cm [37]. Although length at birth will likely depend on maternal length, there are no data to parameterize this relationship for *G*. *melas* and for simplicity we adopted the mean length at birth for both sexes (*l*_*b*_ = 177 cm). Structural length of the fetus (*l*_*p*_) was modeled as a linear function of time since conception (*τ*_*p*_), i.e. $l_{p}=l_{b}\frac{\tau_{p}}{T_{P}}$ for 0≤*τ*_*p*_≤*T*_*P*_ [37]. Accordingly, length at birth was reached when gestation was due (i.e. *τ*_*p*_ = *T*_*P*_). Structural mass followed a power function of structural length: $S(l)=\omega_{1}l^{\omega_{2}}$. Parameters *ω*_1_ and *ω*_2_ were assumed the same for males and females, because male and female long-finned pilot whales were reported to have a similar length-weight relationship and percentage of blubber [34].

Incoming energy was derived from prey feeding (*I*_*R*_) and, for calves, milk consumption (*I*_*L*_). Outgoing energy was spent on field metabolic costs (*C*_*M*_), costs of structural growth (*C*_*G*_), lactation costs (*C*_*L*_ for lactating females) and fetal development costs (*C*_*P*_ for pregnant females). For each whale, reserve mass increased if total incoming energy exceeded total outgoing energy (anabolism) and decreased in the opposite case (catabolism):

$$\frac{dF}{da}\;=\left\{ \begin{array}{ll} \varepsilon_{i}^{-1}(I_{R}+I_{L}-C_{G}-C_{M}) & \mathrm{Calves}\\ \varepsilon_{i}^{-1}(I_{R}-C_{G}-C_{M}-C_{P}) & \mathrm{Pregnant}\;\mathrm{female}\\ \varepsilon_{i}^{-1}(I_{R}-C_{G}-C_{M}-C_{L})\; & \mathrm{Lactating}\;\mathrm{female}\\ \varepsilon_{i}^{-1}(I_{R}-C_{G}-C_{M}-C_{P}-C_{L})\; & \mathrm{Pregnant}\mathrm{and}\;\mathrm{lactating}\;\mathrm{female}\\ \varepsilon_{i}^{-1}(I_{R}-C_{G}-C_{M}) & \mathrm{Other} \end{array}\right.$$ (2)

The parameter *ε*_*i*_ accounted for the efficiency of anabolism (*ε*_+_ if $\frac{dF}{da}>0$) and catabolism (*ε*_−_ if $\frac{dF}{da}<0$) [38].

The rate of prey feeding (*I*_*R*_ in MJ assimilated energy day^-1^) was given by:

$$I_{R}(R,a,S,F,W)=\phi_{R}R\;S^{2/3}\frac{a^{\gamma }}{T_{R}^{\gamma }{+a}^{\gamma }}\frac{1}{1+e^{-\eta (\rho W/F-1)}}$$ (3)

This included a linear response to prey density and structural surface-area (*ϕ*_*R*_*RS*^2/3^; [33]), with attack rate parameter *ϕ*_*R*_. The hyperbolic function of whale age $\frac{a^{\gamma }}{T_{R}^{\gamma }{+a}^{\gamma }}$ modeled a gradual increase in prey feeding efficiency during the lactation period and early juvenile phase, under the assumption that experience in the highly coordinated social hunting strategies of pilot whales comes with age [39, 40]. Within this function, *T*_*R*_ denoted the age at which feeding efficiency equaled 50% and parameter *γ* controlled function shape (non-linearity). The last fraction in Eq (3) is the feeding level and represented the fraction of time spend foraging, which followed a sigmoidal decreasing function of body condition. This function equaled 0.5 at the target body condition threshold *ρ* = 0.3 and approached 1 for individuals with poor body condition. Simultaneously, it simulated a decrease in feeding activity for whales with high body condition to ensure that reserve mass did not grow out of bounds [32, 38]. Parameter *η* controlled the steepness of this function around FW=ρ.

The rate of milk consumption by calves (in MJ assimilated energy day^-1^) was given by:

$$\begin{array}{l} I_{L}(a,S,F,W,F_{m},W_{m})=\phi_{L}S^{2/3}\frac{1}{1+e^{-\eta (\rho W/F-1)}}\times \\ \min\left(1,{\left[\frac{1-\frac{a-T_{N}}{T_{L}-T_{N}}}{1-\xi_{c}\frac{a-T_{N}}{T_{L}-T_{N}}}\right]}_{+}\right){\left[\frac{\left(1-\xi_{m})(F_{m}-\rho_{s}W_{m}\right)}{\left(\rho -\rho_{s})W_{m}-\xi_{m}(F_{m}-\rho_{s}W_{m}\right)}\right]}_{+} \end{array}$$ (4)

where a ‘+’-subscript indicates that only positive values were used, i.e. [*f(x)*]_+_ = max(*f(x)*, 0). Similar to prey feeding, milk consumption was proportional to calf structural surface area (ϕLS23) with proportionality constant *ϕ*_*L*_ and a decreasing sigmoidal function of calf body condition. In addition, calf age influenced the milk assimilation rate according to the minimum function in Eq (4). Beyond age *T*_*N*_, milk suckling rate decreased with age until it reached zero at the age at weaning (*T*_*L*_ = 1223 days ~ 3.35 years). The last factor in square brackets in Eq (4) allowed for regulation of milk provisioning by the mother. This function decreased with body condition of the lactating female (indicated with subscript *m*) and ensured that lactation stopped if her body condition fell below the starvation threshold *ρ*_*s*_ = 0.15. Because the milk assimilation rate of a calf depended on the body condition of its mother, the model kept track of the unique link between each calf and its mother. Parameters *ξ*_*c*_ and *ξ*_*m*_ controlled the shape (non-linearity) of their function components (see S1 File).

The energetic losses through field metabolic rate (in MJ day^-1^) were assumed to follow a ¾ power law of the maintenance body mass (*W*_*M*_), *i*.*e*. $C_{M}=\sigma_{M}W_{M}^{3/4}$, where *W*_*M*_ = *S*+*θ*_*F*_*F* discounted the contribution of reserve mass, which were assumed to have lower mass-specific maintenance costs compared to structural mass (*θ*_*F*_ = 0.2) and *σ*_*M*_ acted as metabolic scalar. For pregnant females, the maintenance body mass also included the structural mass of the fetus, *i*.*e*. *W*_*M*_ = *S*+*θ*_*F*_*F*+*S*(*l*_*p*_). Fetal structural growth costs were accounted for by the costs of gestation (in MJ day^-1^), which followed from the derivative of fetal structural mass with respect to time since conception (*τ*_*p*_), multiplied by a cost of growth parameter *σ*_*G*_, *i*.*e*. $C_{P}=\sigma_{G}\omega_{1}\omega_{2}{\left(\frac{l_{b}}{T_{P}}\right)}^{\omega_{2}}{\tau_{p}}^{\omega_{2}-1}$. Structural growth costs of free-living individuals (in MJ day^-1^) were proportional to the derivative of structural mass with respect to length: ${C_{G}=\sigma }_{G}\omega_{1}k(l_{\infty }-l)\omega_{2}l^{\omega_{2}-1}$. Costs of lactation for lactating females (in MJ day^-1^) were proportional to the milk assimilation rate of their calf: *C*_*L*_ = *I*_*L*_/*σ*_*L*_, with parameter *σ*_*L*_ accounting for efficiency of both milk production and milk assimilation.

At birth every individual was randomly assigned an age at which it died (life-expectancy at birth), which followed the age-distributions of male and female *G*. *melas* in the North-East Atlantic [36]. We used these distributions to estimate age-dependent mortality rates under the assumption that the North-East Atlantic *G*. *melas* population was stationary [41] and starvation-related mortality was low. The age-distribution for calves (both sexes) and weaned females best reflected a Siler mortality rate, *D*_*a*_, that declined with age for calves and young females, reached a minimum around 15 years and increased for older females [42, 43].

$${D_{a}(a)=\alpha }_{1}e^{{-\beta }_{1}a}+\alpha_{2}e^{\beta_{2}a}$$ (5)

**Fig 2:**
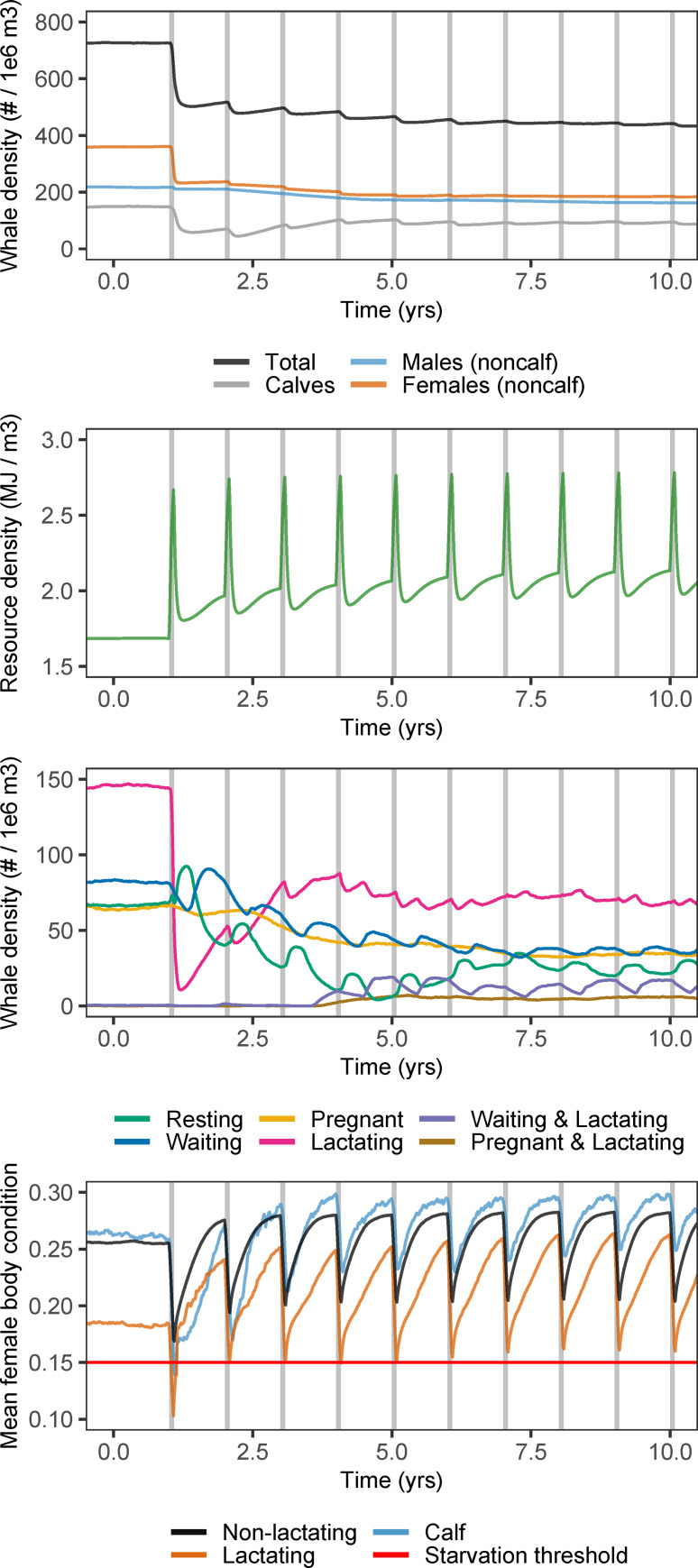
Population response to disturbance. Response of the population at stationary state to the onset of a disturbance corresponding to 30 days without foraging repeating every year. Disturbance periods are indicated with gray shaded bars and the first disturbance event occurred 1 year into the simulation. Preceding 49 simulated years allow the undisturbed population to reach its stationary state and are not shown. All densities represent the mean values of 100 replicate simulations. Bottom panel shows mean body condition of calves (age < 1223 days), lactating females (including ‘waiting and lactating’ and ‘pregnant and lactating’ females) and non-lactating females (‘resting’, ‘waiting’ and ‘pregnant’ females with age > 1223 days) from a single simulation with output collected every 5 days.

Age-distribution of weaned males was best approximated with a constant (age-independent) mortality rate: *D*_*a*_ = *μ*_*male*_. Male mortality exceeded female age-dependent mortality until approximately 32 years of age, when female mortality increased beyond male mortality (Fig 2 in S2 File).

If body condition of an individual fell below the starvation threshold (*i*.*e*. $\frac{F}{W}<\rho_{s}$, with *ρ*_*s*_ = 0.15), it suffered from starvation mortality *D*_*s*_*(F*,*W)*, in addition to age-dependent mortality, where

$$D_{s}(F,W)=\mu_{s}\left(\frac{\rho_{s}W}{F}-1\right)\;\mathrm{if}\;F/W<\rho_{s}.$$ (6)

In our model, starvation did not necessarily lead to immediate death, but it reduced remaining life expectancy by an extent that was an integrated measure of starvation duration (number of days of starvation) and severity of starvation (difference between *F/W* and *ρ*_*s*_). We implemented this by tracking the survivorship (0–1) of each individual: $H(a)=e^{-\int_{0}^{a}(D_{a}(\alpha )+D_{s}(F(\alpha ),W(\alpha )))d\alpha }$, which is a decreasing function of age. An individual died when its survivorship fell below the survival threshold, the uniform random number between 0 and 1 assigned at birth. The life-expectancy at birth was given by the age at death if no starvation was experienced. In case of one or more starvation events, the associated starvation mortality reduced age at death below the life-expectancy at birth, because survivorship dropped below the survival threshold at a younger age. This implementation of starvation-induced mortality allowed for individual-level variation in the outcome of starvation, while retaining computational efficiency. For some individuals, mainly those with a low survival threshold, starvation induced a reduction in remaining life expectancy, while for others it resulted in death.

Reproduction was initiated when absolute reserve mass exceeded the amount of reserves needed to produce a neonate ($F_{neo}=\frac{\sigma_{G}\omega_{1}{l_{b}}^{\omega_{2}}}{\varepsilon_{–}}+\frac{\rho_{s}\omega_{1}{l_{b}}^{\omega_{2}}}{\left(1-\rho_{s}\right)}$) plus the reserve mass threshold below which starvation mortality occurred (*ρ*_*s*_*W*). Pregnancy was therefore triggered by the amount of surplus energy, following Klanjscek et al. [35]. The moment of crossing this ‘pregnancy threshold’ was separated from the onset of pregnancy by a waiting period, the length of which was determined randomly according to an exponential distribution with a mean of 445 days. The waiting period accounted for the frequency of ovulation (assumed once per year), combined with a chance of pregnancy upon ovulation of 0.82 [21, 37]. We did not account for seasonal variation in the chance of pregnancy, since births and ovulations appear to occur year-round [37]. Pregnancy started when the waiting period was completed, irrespective of the reserve mass at that point in time, and lasted one year [37]. Termination of pregnancy either as a result of fetal mortality or abortion was not considered. Termination of pregnancy in response to female condition is considered in several density-independent bio-energetic models of cetaceans [44–46] and would reduce disturbance effects on female mortality and increase reproductive output by providing earlier re-initiation of pregnancy. However, these effects will likely only play a minor role in our density-dependent model setting, because the average daily costs of pregnancy are relatively low compared to those of lactation [21]. For simplicity, fetuses were assumed to have no reserves. At birth, each fetus obtained an amount of reserve mass from its mother such that its body condition equaled *ρ*_*s*_, *i*.*e*. $F=\frac{\rho_{s}S_{b}}{1-\rho_{s}}$, with $S_{b}=\omega_{1}l_{b}^{\omega_{2}}$ being structural mass at birth. Lactation lasted 1223 days (~3.35 years) and weaning occurred at a length of 292 cm. Lactation stopped if either the calf or the female died. Because lactation rate was modeled as an emergent process that depended on the energetic requirements and body condition of both calf and female, the realized lactation rate could approach zero before weaning if the calf was able to cover all its energy demands by prey feeding, or if its mother’s body condition fell below *ρ*_*s*_ and she ceased milk supply. Simultaneously lactating and pregnant female pilot whales have been observed [37], so we allowed lactating whales to enter the waiting period during the last year of lactation. This prevented females from having two calves simultaneously. Non-pregnant and non-lactating females that had not entered the waiting period were categorized as ‘resting’. Thus, at any point in time a female whale could be in one of the following reproductive states: pregnant; lactating; lactating and waiting; lactating and pregnant; waiting; or resting.

### Disturbance

As in Hin et al. [21], disturbance was modeled as a yearly recurrent period of consecutive days of no foraging affecting all individuals in the whale population. Cessation of feeding could for example occur when individuals are displaced from prey-rich feeding habitats. A disturbance event was characterized by a starting date (*t*_*start*_; day within each year) and a disturbance duration (*t*_*dist*_; number of consecutive days). During a disturbance event, prey feeding of all individuals was set to zero for 24 h per day and compensatory feeding was only possible when disturbance had ended. Hence, disturbance affected the prey attack rate *ϕ*_*R*_ in Eq (3) as

$$\phi_{R}=\left\{ \begin{array}{cc} 0 & \mathrm{if}((t\;\mathrm{mod}\;365)\geq t_{start})\&(\left(t\;\mathrm{mod}\;365)<(t_{start}+t_{dist}\right))\\ 1 & \mathrm{otherwise} \end{array}\right.$$ (7)

Lactation during disturbance was possible. Real-world disturbances are almost certainly less extreme, might only affect a subset of the population and will probably not result in the loss of multiple, consecutive days of foraging. We have purposely choose an extreme disturbance scenario to get an idea of the full scope of potential effects of disturbance on the modeled population. In addition, we assume that disturbance does not negatively affect the prey field.

### Model analysis

We used the Escalator Boxcar Train software package (EBT; [47] available at https://staff.fnwi.uva.nl/a.m.deroos/EBT/Software/index.html) to simulate prey dynamics and the state variables of each individual whale. The integration of these ODEs was interrupted by events of birth, weaning, onset of reproduction (crossing of pregnancy threshold and start of waiting period) and initiation of pregnancy (end of waiting period). These events also determined female reproductive status. Prey density followed from the integration of the ODE in Eq (1). We used R software (v.4.0.2) for handling and plotting of model output [48]. Code to run the model is available online (*link to online repository*).

Here we report on the effect of an increase in disturbance duration (*t*_*dist*_) on the whale population and its prey in the absence of seasonality in prey productivity (*A =* 0). First, we illustrate the effect of density dependence through prey depletion on whale vital rates. Next, we present the response to the onset of yearly repeating disturbances of 30 days per year on an undisturbed whale population at carrying capacity. Then we show how the density of a stationary whale population depends on *t*_*dist*_ and we examine the distributions of body condition and patterns of (age-specific) survival and reproductive output of females from these stationary populations. We assessed robustness of our results to seasonality in prey productivity (*A* = 0.25) and timing of disturbance, which was only relevant in seasonal environments. In this case, we arbitrarily called the season of high and low productivity ‘summer’ and ‘winter’, respectively. We conducted an analysis of the sensitivity of our results to the parameters whose values had little or no empirical support (see S2 File).

## Results

### Density-dependent population dynamics

In absence of disturbance, the whale population initially grew exponentially and depleted the prey base until it reached a stationary state (Fig 1). During population growth, prey depletion affected individual-level energetics, which led to changes in vital rates: calf survival and the fraction of pregnant females decreased, and age at first reproduction increased (Fig 1). At the population level, the fraction of females that were simultaneously ‘waiting and lactating’, or ‘pregnant and lactating’ was reduced to almost zero when the stationary state was reached. At the stationary state, mean female lifetime reproductive output equaled 1.

### Population response to disturbance

The onset of disturbance in an undisturbed population at stationary state led to a sharp decline in population density and a peak in prey density due to the lack of prey foraging by whales (Fig 2). The initial population decline was mainly attributed to a drop in the number of lactating females and calves. During the first disturbance event, mean body condition of lactating females dropped below the starvation mortality threshold, indicating that the decrease in the number of lactating females was driven by starvation-induced mortality. The decrease in mean body condition of calves was larger than that of lactating females but did not reach as low levels because calf body condition was much higher pre-disturbance (Fig 2).

After the first disturbance event, whale population density continued to decline more gradually over subsequent disturbance events until it reached a new stationary state. The decline of whale density was paralleled by an overall increase in prey density, with yearly peaks during each disturbance event. Once the number of lactating females recovered from the initial disturbance episode, some lactating females were observed that were simultaneously ‘waiting’ or ‘pregnant’, while these were absent in the undisturbed population. At the new stationary state, disturbance events led to changes in the distribution of female reproductive classes and decreased mean body condition, but no longer affected whale density. Irrespective of age and reproductive status, mean body condition increased in between disturbances beyond its pre-disturbed level. This overall increase was such that mean body condition stayed well above the starvation threshold during later disturbance events.

### Effect of disturbance duration on population density

**Fig 3:**
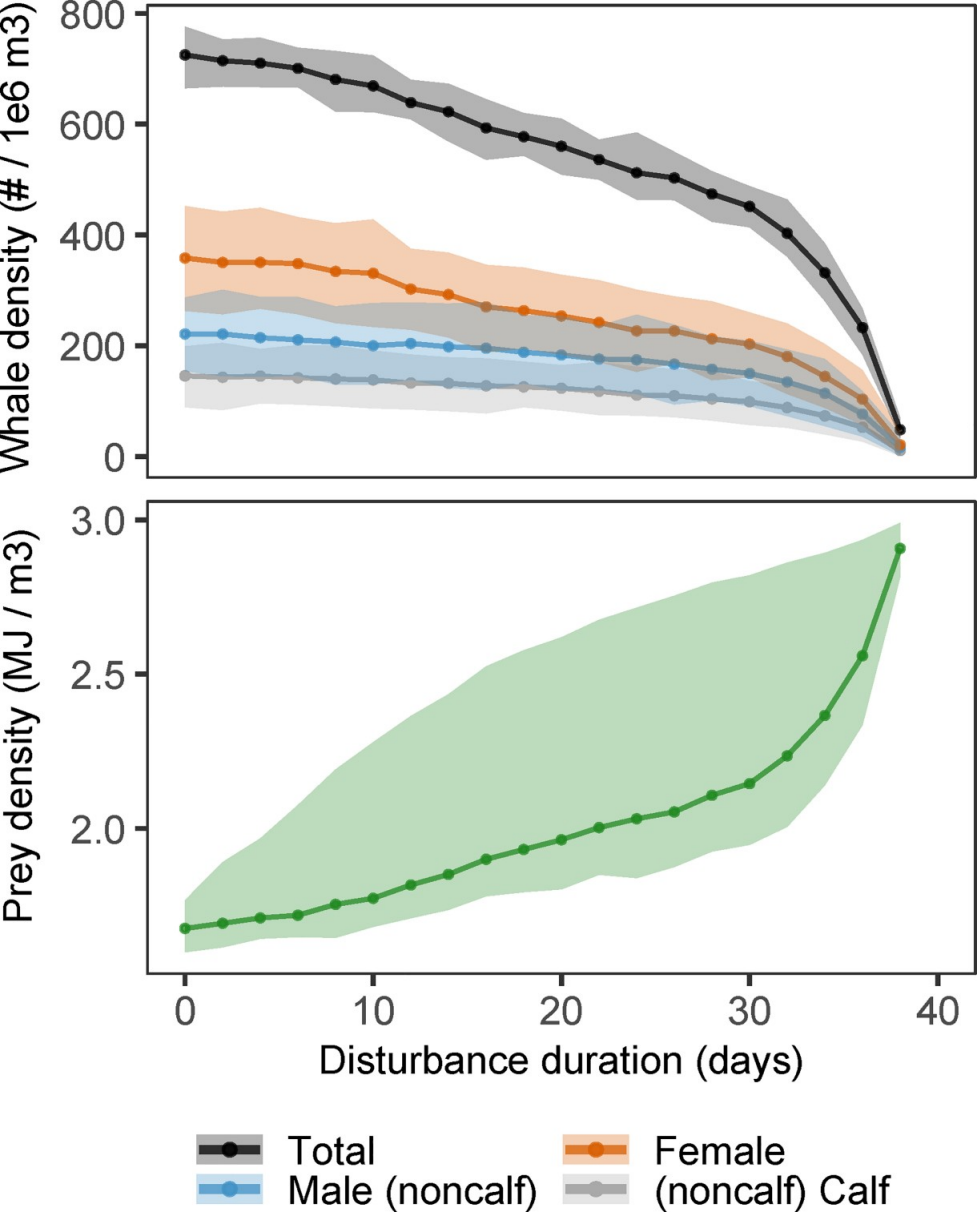
Effect of disturbance duration on population density. Whale (top panel) and prey density (bottom panel) in the stationary population as a function of disturbance duration (*t*_*dist*_). Each point represents the time-averaged density of 200 simulated years, after an initial 200-year period to allow the population to reach its stationary state. Output was collected every 5 days (14600 observation per point). Lines connect mean densities and shaded areas indicate min/max density.

Whale density in the stationary population decreased in a non-linear manner as a function of the duration of the yearly recurrent disturbances (Fig 3). Mean whale density decreased by 54% as disturbance duration increased from 0 to 34 days per year, while the population went extinct when disturbance lasted 6 days longer. Overall, longer yearly disturbances decreased top-down control by whales, leading to an increase in mean prey density (Fig 3). The increased variation in prey density was caused by the cessation of foraging during each disturbance event that led to yearly peaks in prey density (Fig 2), whose amplitude was highest for intermediate disturbance durations.

### Body condition

**Fig 4:**
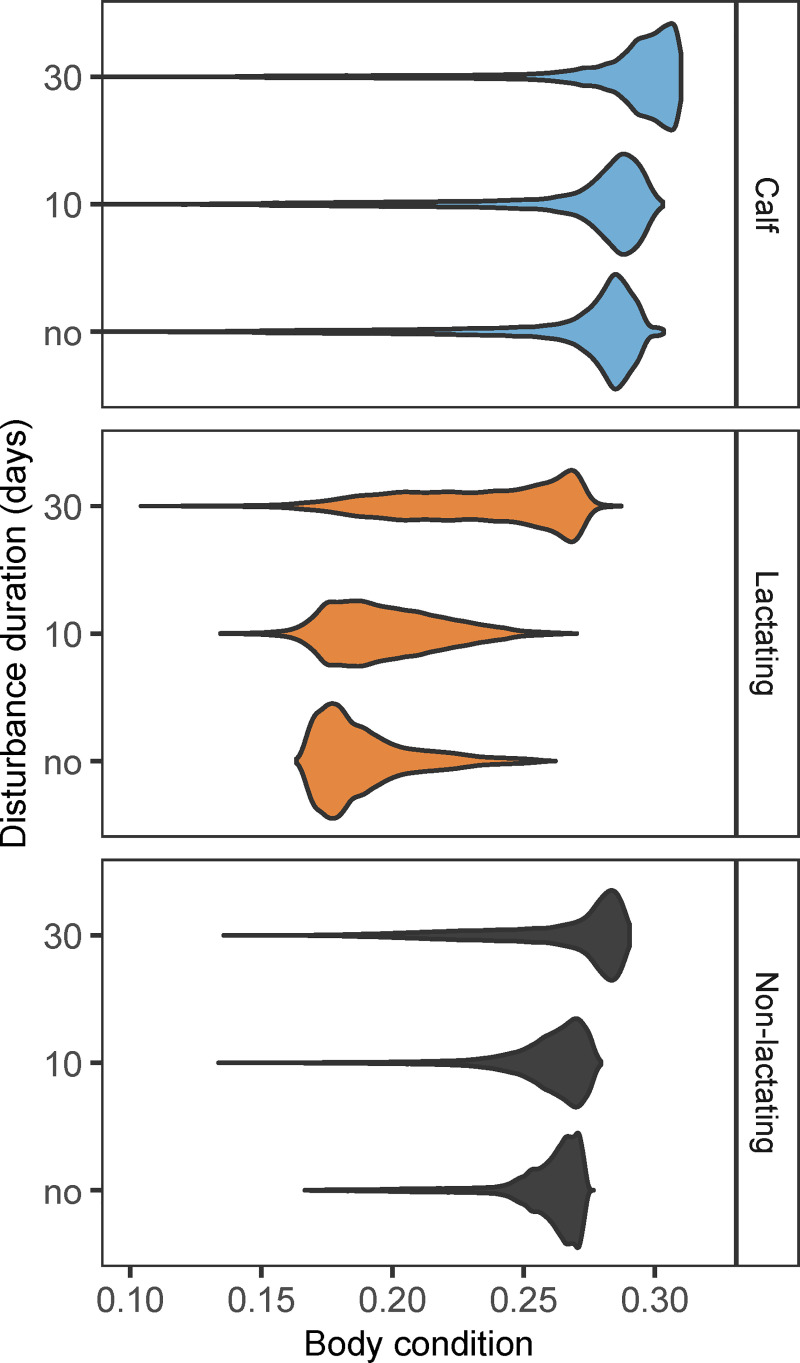
Effect of disturbance duration on body condition. Distributions of body condition per reproductive class for different disturbance durations (*t*_*dist*_). Calves (age < 1223 days) are plotted as a separate reproductive class. Females that are simultaneously lactating and waiting or lactating and pregnant are referred to as lactating. Females that were resting, waiting or pregnant are collectively referred to as non-lactating. Data in each panel is derived from a simulation of a stationary population over 100 years with data collected every 10 days for 1000 randomly selected females, after an initial transient period of 100 years. Each distribution has a surface area of 1.

Body condition of females in a stationary population was highest for calves and lowest for lactating females, irrespective of disturbance duration (Fig 4). Resting, waiting and pregnant females (collectively referred to as ‘non-lactating’ in Fig 4) had similar body conditions, although body condition was generally highest in waiting females and lowest in resting females. Although disturbance induced fluctuations in body condition over time (Fig 2), females that lived in disturbed populations had an overall higher body condition than females from undisturbed populations (Fig 4). Improvement in body condition occurred because of the increase in prey density associated with disturbance (Fig 3), which increased per capita prey availability. Higher prey density led to a higher feeding rate and increased energy reserves of individual whales. The improvement in body condition occurred irrespective of reproductive status and was largest for lactating females, as their normally poor body condition allowed more scope for improvement (Figs 2 and 4). Disturbance also increased the variation in body condition, especially for non-calf individuals. In populations subject to 30 days of disturbance, some lactating females, mostly young animals that were still growing, had a poor body condition.

### Age-specific survival and reproduction

**Fig 5:**
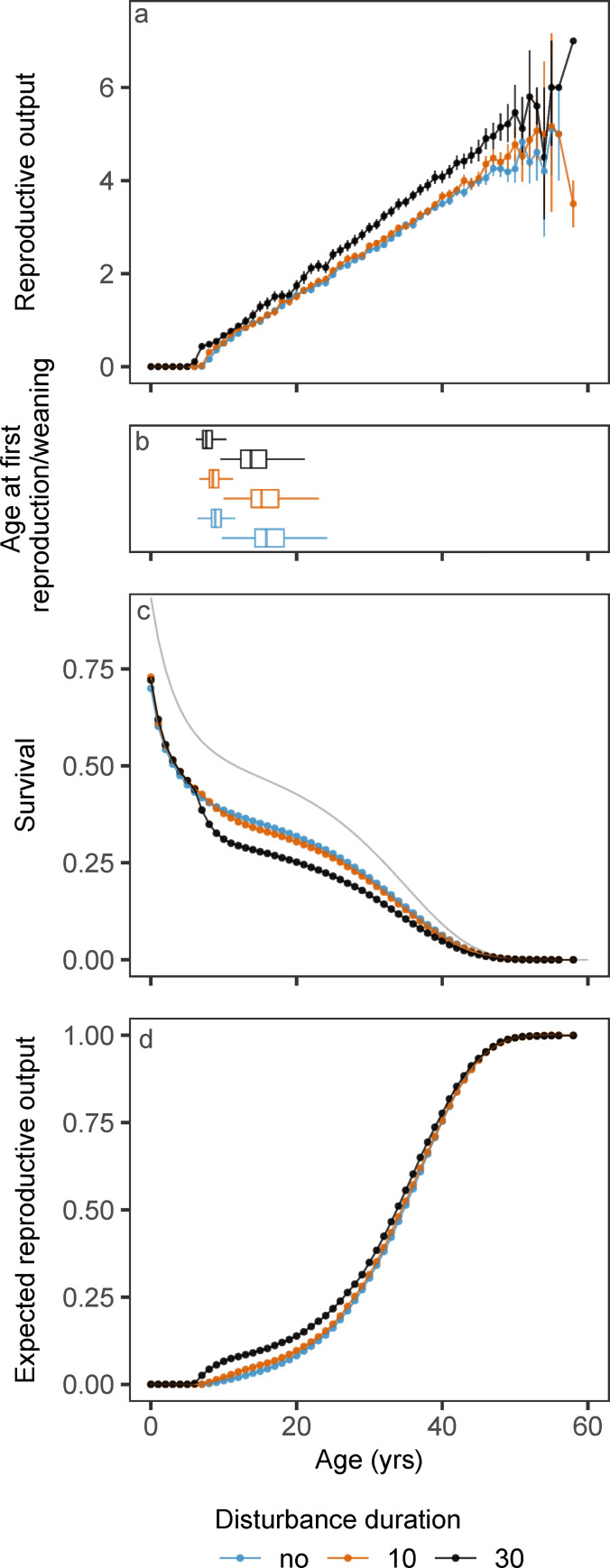
Age-specific survival and reproduction. Age-specific patterns of survival and reproduction for different disturbance durations (colors). Panel a): the cumulative mean number of female calves born per female age class (bin size of 1 year). Error bars represent 95% confidence intervals of the means derived from non-parametric bootstrapping. Confidence intervals increase with age due the lower number of females in older age classes. Panel b): female age at first reproduction (left-most boxplots) and female age at weaning of first calf (right-most boxplots). Boxes show the 25% quantile (left end), median (vertical bar) and 75% quantile (right end). Whiskers extend 1.5 times the inter-quartile range beyond the edges of the box, or indicate extreme values if these fall within that range. Panel c): age-specific survival calculated as 1 minus the cumulative proportion of females that died within each year class. The gray line represents the survival in absence of starvation mortality (only age-dependent mortality), which is given by: $e^{-\int_{0}^{\alpha }D_{a}(a)da}$. Panel d): the age-specific product of survival (c) and the cumulative reproductive output (a). Over the entire lifetime of a female, this measure should approach 1 (*R*_*0*_: the expected lifetime reproductive output) in a population at stationary state.

The pattern of age-specific survival and reproduction of females in stationary populations changed in response to disturbance duration (Fig 5). The mean number of female calves born to a single female was higher in disturbed populations, across all female ages (Fig 5A). Because females from disturbed populations were generally in better condition, these females crossed the pregnancy threshold at a younger age and reproduced earlier (Fig 5B). Mean age at first reproduction (AfR) was more than one year earlier if the population was disturbed for 30 days per year, compared to a non-disturbed population. Earlier reproduction of females in disturbed populations also translated into a reduction in the female age at weaning of her first calf (AfW; Fig 5B). At 30 disturbance days per year, mean AfW was on average over 2 years earlier compared to the undisturbed case.

There was significant starvation-induced mortality of calves, mainly during the first year of life, across all disturbance scenarios (including no disturbance; Fig 5C). In Fig 5C, the thin gray line represents the survival curve in absence of starvation mortality (only age-dependent mortality), which is given by $e^{-\int_{0}^{\alpha }D_{a}(a)da}$. Already during the first year, survival was reduced below this curve. Without disturbance, 47% of the whales died while they were still calves (age < 1223 days), and around 50% of those calves experienced starvation-induced mortality that almost always (99.7%) led to immediate death. Density dependence thus reduced calf survival (Fig 1) at high population density, and this effect was largely unaffected by disturbance.

High levels of disturbance affected adult female survival. With 30 days of disturbance, starvation-induced mortality occurred among young mature females that were raising their first calf, resulting in a drop of age-specific survival around years eight to ten compared to no disturbance or ten days disturbance per year (Fig 5C).

The expected cumulative reproductive output up until a certain age represents the joint outcome of reproduction (counting female calves only) and survival and increased with age to a value of one (expected lifetime reproductive output; Fig 5D). Because the expected reproductive output was higher in disturbed populations and survival was approximately equal during the first years of life across all scenarios, the expected reproductive output increased more rapidly with age compared to the undisturbed case (Fig 5D). This initial difference disappeared at older ages due to the decreased survival of mature females at longer disturbance durations.

### Life expectancy and mean reproductive output

**Fig 6:**
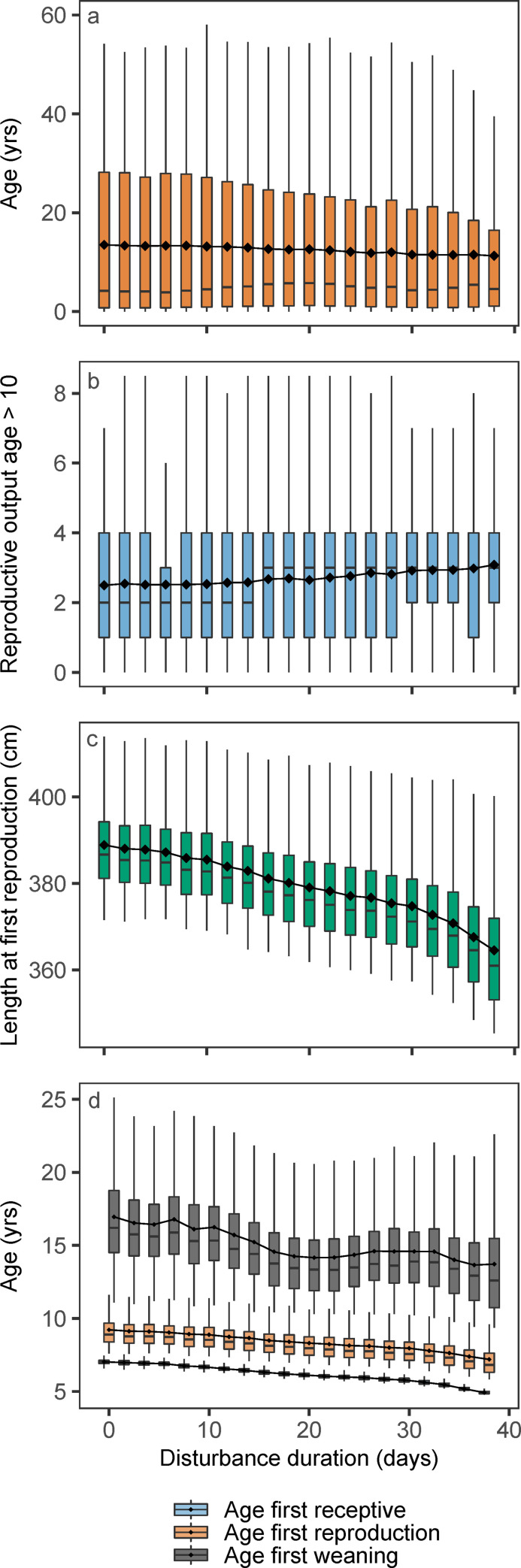
Vital rates. Distribution of a) life expectancy; the mean female age at death, b) lifetime reproductive output for females older than 10 years, c) female length at first reproduction and d) female age at first receptive, first reproduction and weaning of first calf as a function of disturbance duration. Boxplots show medians (horizontal bars) and 25% and 75% quantiles (box edges). Whiskers extend 1.5 times the inter quartile range beyond the edges of the box, or indicate extreme values if these fall within this range. Diamonds and connecting lines show means of each distribution. The color legend applies to bottom panel only.

Longer disturbances led to a slightly lower life expectancy, represented by the mean age at death of females from a stationary population subject to a certain disturbance regime (Fig 6A). Life expectancy for a female born in an undisturbed population was 13.5 yrs and decreased to 11.3 yrs. with 38 consecutive days of disturbance per year. Judging from the age-specific survival pattern (Fig 5C), the decrease in mean life expectancy mainly resulted from increased mortality among females that were lactating their first calf. Mean lifetime reproductive output of females that survived beyond 10 yrs of age increased with disturbance duration from a mean of 2.5 female calves born per female in an undisturbed population to 3.08 female calves at 38 days of disturbance (Fig 6B). In an undisturbed population, females successfully weaned their first calf at a mean age of 17.0 yrs. With 38 days of disturbance mean AfW decreased to 13.7 yrs. Mean age at first receptive and first reproduction followed a similar pattern (Fig 6D). The response of length at first reproduction to disturbance mirrored those of female age at first reproduction (Fig 6C).

### Parameter sensitivity

Results with seasonally varying prey productivity were broadly similar and are described in detail in S2 File. Overall, results were robust to changes in the values of parameters that had little or no empirical support (Figs 9–19 in S2 File). The parameters that modified the effect of whale age on resource assimilation (*γ* and *T*_*R*_) resulted in higher prey density and lower whale density (Figs 9 and 10 in S2 File) when they decreased resource feeding efficiency at weaning. Changes in *T*_*R*_ also resulted in a female-biased population sex ratio at low disturbance durations. Parameters that modified the relationship between whale age and milk feeding efficiency (*ξ*_*c*_ and *T*_*N*_) had a negligible effect on the relation between whale and prey density and disturbance duration (Figs 13 and 14 in S2 File). The effects of parameter changes on whale and resource density were greatest at high disturbance duration. In some cases, this affected the disturbance duration at which the whale population went extinct.

Overall, varying these parameters did not affect the relationships between life history statistics and disturbance duration. For all parameter combinations, mean AfR, mean AfW and female life expectancy decreased with increasing disturbance duration (Figs 16–18 in S2 File), while mean lifetime reproductive output of females beyond age 10 yrs increased (Fig 19 in S2 File).

## Discussion

We used a density-dependent, individual-based population model to investigate how non-lethal disturbance affects individual body condition (fraction of energy stores to total body mass), vital rates and population density for a medium-sized cetacean. The undisturbed whale population was regulated through the effect of prey availability on calf survival and pregnancy rates and on age at first reproduction of females. Disturbance decreased whale population density and increased prey availability. This led to a decrease in age at first reproduction and increased mortality among young females that were raising their first calf. However, females from populations subject to yearly repeating disturbances had, on average, improved body condition and higher age-specific reproductive output. Considering the joint effect of disturbance on survival and reproduction, there was no net effect on expected lifetime reproductive output of females (*R*_*0*_), as disturbance merely modified the way in which density dependence balanced survival and reproduction to obtain a value of *R*_*0*_ equal to 1.

Responses to disturbance are mediated through the overall increase in prey availability that resulted from the reduction in top-down prey control (Figs 2 and 3). Higher prey availability increases body condition and the ability to survive prolonged periods without feeding. Because reproduction is initiated when a female’s energy reserves exceed a predefined threshold, individuals with more reserves will on average start reproducing at a younger age. In an undisturbed environment, the mean age at which females first crossed the pregnancy threshold was 7.0 yrs, well within the age-class range for first ovulation of 6 to 9 years as reported by Martin and Rothery [37]. This age decreased to a minimum of 4.9 yrs with 38 days of disturbance. The improvement in body condition in disturbance scenarios also decreased the interval between reproductive attempts (the inter-birth interval). Both effects increased lifetime reproductive output for females that survived beyond 10 years of age.

However, there is a limit to which increased prey availability can offset the increase in starvation-related mortality induced by disturbance, because the upper limit to an individual’s body condition also sets an upper limit to the starvation period that it can survive. This limit depends on an individual’s age and reproductive status (lactating versus non-lactating). Young lactating females are especially vulnerable because they are still growing in structural size and the maximum absolute size of their energy store is therefore smaller. As a result, they suffered increased mortality with longer disturbance durations. During lactation their body condition is also lower compared to that of fully-grown females in the same state of lactation. Disturbance therefore increased starvation-related mortality among these young lactating females, even though reproduction was initiated based on absolute surplus energy (instead of body condition) and milk provisioning stopped when body condition dropped below the starvation threshold. As disturbance duration was increased, the starvation period for young females was prolonged, which ultimately caused the population to decline towards extinction.

Our results have important implications for the management and monitoring of long-lived organisms that are affected by non-lethal disturbances. Counter-intuitively, our results indicate that non-lethal disturbance can increase individual body condition and reproductive output if the population is regulated by density dependence acting through prey depletion. Managers of wildlife populations need to be aware of the potential counter-intuitive responses caused by compensatory changes in prey availability following the onset of disturbance.

According to our results, population density appears to be the only statistic that can reliably indicate that a population is suffering from the effects of disturbance. However, this conclusion should be used with caution, because of the non-linear response of population density to disturbance duration. In addition, one set of traits that are negatively related to fitness (AfR, AfW and length at first reproduction–Fig 6C and 6D) did decrease in a consistent way with increasing disturbance duration and decreasing population density. Similar changes were observed in AfR for baleen whales in the Antarctic during the period 1930–1960 (see Fig 6 of [49]), when population size and average length also declined [10].

Besides changes in mean traits, there is an opportunity to monitor for changes in trait variance. Our results show increased variation in body condition of females from disturbed populations, especially for lactating females (Fig 4). This increased variation results from large annual fluctuations in mean female body condition driven by the recurrent disturbance events (Fig 2). The occurrence of a large number of individuals in particularly poor conditions at certain times of the year might act as a warning signal to indicate that a population is suffering from disturbance. Moreover, effects of disturbance are most severe right after the onset of the first disturbance event, when there is a mortality peak among lactating females and calves (Fig 2). This mortality peak could potentially be picked up by monitoring programs of sufficient scale and intensity. The lethal consequences of disturbance fade as whale and prey densities transit to a new stationary state, which might take several years. This chain of events suggests that the temporal window of any monitoring program is crucial to get a comprehensive picture of how wildlife populations respond to disturbance. Ideally, monitoring should start well in advance of any planned disturbances [8, 9].

Observational and experimental studies of changes in fitness-related traits in relation to density changes have demonstrated that a decrease in population density is associated with a decrease in mean body length in response to size-selective harvesting [10] or experimental reductions in food availability [12]. Our model suggests that non-lethal disturbance that results in a reduction in population density and an increase in prey availability actually leads to an increase in individual body condition, a common used fitness-related trait in cetaceans [34, 50–55]. In our model, we focused on body condition because structural length was a function of age only and growth in structural size did not depend on prey availability. Future studies should provide a more comprehensive understanding of how mean structural length is likely to be affected by disturbance if growth in structural length depends on prey intake rates. Although body size growth in (marine) mammals is generally considered to be demand-driven, variation in length-at-age could occur as a result of stunted growth from malnutrition, which is well-documented in humans [56] and likely applicable to cetaceans too.

Unsurprisingly, the type of sublethal disturbances that we simulated, a continuous, multi-day period of no feeding, had lethal consequences for the subset of the population that suffered highest energetic costs and had lowest reserve capacity. The effect on survival will likely be reduced if disturbances are more sporadic or impact only a subset of the population. In such case, there might be no differences in the rates of reproduction and survival between disturbed and undisturbed populations at stationary state, but only an effect on individual body condition and population density. The lower stationary population density under disturbance would then be driven solely by the higher prey density that a female would require to produce one female offspring on average. This is different from the transition phase, as the decrease of the population following the initial onset of disturbance will be driven by temporary changes in survival and reproduction, given that immigration and emigration are not considered in this model. How the response to disturbance depends on the disturbance scenario used is a pivotal topic for future research for which the current model is well-suited.

Bio-energetic models have been used to investigate the potential effects of disturbance on vital rates in gray whales (*Eschrichtius robustus* [57, 58]), blue whales (*Balaenoptera musculus* [20, 59]), elephant seals (*Mirounga leonina* [6, 60]), humpback whales (*Megaptera novaeangliae* [61]), sperm whales (*Physeter macrocephalus* [44, 45]) and long-finned pilot whales [21]. In all these studies, disturbance was assumed to result in lost energy intake or increased energetic expenditure. These studies showed how reduced energy intake can lead to a prolonged reproductive cycle, decreased calf survival, or reduced post-weaning survival, either through reduced calf mass at weaning or early calf weaning. If energy intake is reduced further, female survival is affected. For pilot whales, Hin et al [21] showed that disturbance in a density-independent setting increased mortality of calves born to young females, which increased the age at which females weaned their first calf and decreased *R*_*0*_. These life history responses to disturbance are in contrast to those reported here. The difference is entirely attributable to the effect of density dependence.

Marine mammal populations are generally assumed to be regulated by density-dependent processes [62, 63] and density dependence is fundamental to the provisions of the US Marine Mammal Protection Act [64], and the Revised Management Procedure used by the International Whaling Commission [65]. With the exception of colonially breeding pinnipeds, where crowding and availability of suitable breeding sites may limit population size [66], most populations of marine mammals are assumed to be limited by prey / resource availability [25, 26, 67]. For example, Laws [68] attributed the multiple changes in the demographic rates of large baleen whales in the Southern Ocean observed during the period of intensive commercial whaling to a massive reduction in the consumption of krill (*Euphausia supurba*) by these species.

For mammals, most evidence of density effects on individual vital rates (*i*.*e*. survival and reproduction) or fitness-related traits (*e*.*g*. body size / condition) comes from terrestrial mammal populations. In ungulates, increased density is correlated with a reduction in adult body mass [69], a decrease in juvenile survival and an increase in age of first reproduction [70–72]. A review on both large terrestrial herbivores and marine mammals showed that in 66 studies (36%), first-year survival was the most important density-dependent demographic parameter [73]. For North Atlantic fin whales (*Balaenoptera physalus*), Williams et al. [50] showed that *per capita* plankton availability—which was inversely related to fin whale abundance–was positively related to blubber thickness, which in turn determined fin whale pregnancy rates. Density effects on female pregnancy rate, calf survival and age at first reproduction were produced by our individual based population model (Fig 1), indicating that our model was able to capture some aspects of the dynamics of real-world populations. In addition, we have shown that density dependence can systematically alter the response of populations to disturbance and this calls for a better understanding of the processes that regulate natural populations of these long-lived animals.

A recent analysis of survey data for North-East Atlantic long-finned pilot whales revealed that the population has remained stable during the period 1987–2015 [41]. Although this corresponds to our assumption of a stationary population at carrying capacity, it is currently unclear to what extent processes other than prey availability are responsible for density regulation in this population. Many populations of marine mammals are faced with a multitude of human-induced stressors [74–76] that might reduce their density below the carrying capacity set by their environment. The way in which different stressors (*e*.*g*. noise disturbance, pollutants, harvesting) interact to affect wildlife populations is an actively developing area of research [15, 20, 45].

Further examination of the extent to which our results are contingent upon modeling assumptions that affect the strength of the predator-prey coupling is required. In our modeled system there is strong predator-prey coupling and the predator is assumed to be at a carrying capacity determined by the productivity of its prey. In this setting, increased disturbance-induced mortality among a subset of the whale population leads to reduction of top-down control and increased prey density. Real marine systems possess many features that might weaken the interaction between predator and prey, such as spatio-temporal variability in prey productivity or seasonal movement of the predator. Also, competition by other predatory species can prevent pilot whales profiting from increased prey availability in post-disturbance periods, and thus reduce the potential for compensatory feeding. Furthermore, if disturbance also affects the prey field, by causing prey movement, scattering of dense prey patches or prey mortality [77–79], the potential for compensatory effect of disturbance will likely be reduced. Lastly, the pilot whale foraging model described in Eq (3) assumes that prey intake rate is a linear function of prey density, although whales in good condition will reduce foraging effort. This linear functional response implies that the prey intake rate of whales in poor body condition is not limited by the time required for prey handling or searching. Incorporating more realistic activity budgets that capture the multitude of physiological constraints on foraging behavior of deep-diving cetaceans [80, 81] will likely reduce the ability of individuals to regain lost reserves. Further work that addresses these complexities will be required to obtain a more comprehensive understanding of the population consequences of disturbance in a density-dependent setting. Nevertheless, a decrease in population density due to non-lethal disturbance in a density-dependent setting is bound to translate into some positive, compensatory effects on individual performance, regardless of the exact mechanism by which such density dependence operates.

## Supporting information

S1 File Model parameterization. (DOCX)

S2 File Supplemental results. (PDF)
