# Supplementary material for: Density dependence can obscure nonlethal effects of disturbance on life history of medium-sized cetaceans
Source: PLoS One. 2021 Jun 3;16(6):e0252677. doi: 10.1371/journal.pone.0252677 (PMC8174747; doi:10.1371/journal.pone.0252677)
Supplement: S1 File — (DOCX) [file pone.0252677.s001.docx]

S1 File. Model parameterization

Supplement to

“*Density dependence can obscure nonlethal effects of*

*disturbance on life history of medium-sized cetaceans*”

# Submitted to PlosONE

Model parameters and equations are summarized in Tables 1 and 2, respectively. Most parameter values were derived from a report of the International Whaling Commission on the biology of northern hemisphere long-finned pilot whales, *Globicephala melas* [1].

*Structural growth*

To estimate the Von Bertalanffy growth rate (*k*) and asymptotic length ($l_{\infty}$), we calculated non-linear least squares estimates for males and females on the mean length at age data in Table 10 of [2]. These data were combined with the best estimate for the length at birth (177 cm) given by [3], which did not differ significantly between sexes. As such, the Von Bertalanffy functions for males and females were constrained at (*a,l*) = (0,177). Since age class *i* included all individuals with age (in years) between *i* and *i*+1, we applied an age transformation of 0.5 to obtain mean values for each age class. This resulted in satisfactory fits of the Von Bertalanffy equations (Figure 1 in S2 Supplemental results). Estimates for *k* were divided by 365 to obtain growth rates per day.

Data on body weight and relative contribution of lipids to total body weight where used to estimate the structural length-weight parameters $\omega_{1}$ and $\omega_{2}$ and the target and starvation body condition parameters $\rho$ and $\rho_{s}$. Best estimate for mass at birth was 75 kg [2] and lipid proportion for calves was 17% [4]. Combining these gives a structural mass at birth of *S_b_* = 75 (1 – 0.17) = 62 kg. However, most lipids of calves are probably of structural origin and cannot be used as an energy reserve without compromising survival. To account for this, we set the starvation body condition threshold $\rho_{s}$ to 0.15. Bloch et al [2] report ultimate body weight for female pilot whales of 1320 kg at a length of 512 cm. Of these individuals, lipid proportion is around 25% [4], leading to an ultimate structural mass of $S_{\infty}=990$ kg. Combining $S_{\infty}$ and *S_b_* gives a structural length-weight scaling exponent of: $\omega_{1}={\ln\left( \frac{S_{\infty}}{S_{b}} \right)}/{\ln\left( \frac{l_{\infty}}{l_{b}} \right)}=2.6$, with a scaling constant of $\omega_{2}=8.5\cdot{10}^{-5}$. According to Table 3 in [4], length-weight relationships and percentage of blubber are not significantly different between males and females. Therefore, we adopt the structural length-weight relationship derived for females for males also. This implies that males reach an asymptotic structural mass of 8.5 · 10^-5^ · 594^2.6^ = 1384 kg and asymptotic total mass of 1845 kg. This compares reasonably well with the asymptotic weight of 1959 kg predicted from the Laird-Gompertz growth curve in [2]. The target body condition parameter $\rho$ sets the body condition at which individuals are feeding at 50% of their maximum feeding effort. Because in constant environments the body condition reaches an equilibrium at values below $\rho$, we set $\rho=0.3$, which is slightly above the observed lipid proportion of fully-grown pilot whales.

## Metabolic parameters

The metabolic rate scalar $\sigma_{M}$ was set at 0.75, which represents 2.5 times basal metabolic rate as given by [5] and adopted by [4] for pilot whales. The metabolic rate scalar is applied to the maintenance body weight, which is a weighted sum of structure and reserve mass, with weighting factors 1 and $\theta_{F}$, respectively. We account for relatively low contribution of energy stores (reserves) to maintenance ($\theta_{F}=0.2$) to account for the increase in locomotion and activity costs due to increased drag.

Parameter $\sigma_{G}$ represents costs of structural growth and includes growth overheads and energy in newly synthesized tissue. A newborn pilot whale of 75 kg has an energy density of 7.95 MJ/kg [4] and total energy content of 7.95 · 75 · 1.25 = 745 MJ when accounting for a placenta that comprises 25% of neonate mass. Using Brody’s [6] equation ($Q_{G}=4400{W(0)}^{1.2}$ in kCal), the heat of gestation for a 75 kg neonate equals 3274 MJ. This includes fetal maintenance rate during the gestation period, which in our model amounts to: $\int_{0}^{T_{P}} \sigma_{M}\left( \omega_{1}\left( l_{b}\frac{\tau_{p}}{T_{P}} \right)^{\omega_{2}} \right)^{0.75}d\tau_{p}=1987$ MJ. Combining these number gives a growth efficiency for fetuses of 745 / (3274 – 1987 + 745) = 0.366. Applying this growth efficiency to the energy density of pilot whale calves (10.1 MJ/kg [4]), gives an estimate of $\sigma_{G}=27.4$ MJ/kg. Because Brody’s equation likely underestimates the heat of gestation, we adopt $\sigma_{G}=30$.

According to Lockyer [4], the efficiency by which calves assimilate milk is 0.95 and the efficiency at which the mammary gland produce milk is 0.9. We multiply these to obtain our value for the efficiency of lactation: $\sigma_{L}$ = 0.86.

The efficiency of catabolic reserve dynamics ($\varepsilon_{-}$) was derived from the energy density of lipids (40 MJ/kg; [4]) and assuming a catabolic conversion efficiency of 0.9, which leads to $\varepsilon_{-}$= 35 MJ/kg. Anabolic conversion was assumed to be less efficient and we set $\varepsilon_{+}$ to 55 MJ/kg [7].

According to Lockyer [4], pilot whale calves are fully dependent on milk in their first year of life. We use this as a basis to derive our estimate for the lactation scalar $\phi_{L}$. During the first year, pilot whale calves spend $\int_{0}^{365} \sigma_{M}\left[ \left( 1+\theta_{F}\frac{\rho}{1-\rho} \right)\omega_{1}{l(a)}^{\omega_{2}} \right]^{0.75}da=7808$ MJ on maintenance costs (assuming $F/W=\rho$). Using the Von Bertalanffy length-age relationship and the parameterized structural length-weight relationship, first-year growth equals 41 kg, which costs 1230 MJ. Mean structural mass during this period is $S=\left( \int_{0}^{365} \omega_{1}{l(a)}^{\omega_{2}}da \right)/365=80.5$ kg. Adopting again $F/W=\rho$ for both mother and calf, the milk ingestion rate function simplifies to: ${(7808+1230)}/{365}=\phi_{L}{\cdot80.5}^{2/3}\cdot0.5$, which gives $\phi_{L}=2.7$.

*Time constants*

According to [3], duration of gestation and lactation are 365 and 1223 days, respectively. Parameter *T_N_* modulates the onset of the decrease in milk assimilation with calf age and was set to 365 days following [4]. The time delay between crossing the pregnancy threshold and the onset of pregnancy follows an exponential distribution with rate parameter $\psi= 0.00225$. This corresponds to a mean waiting period of 445 days, which was derived from assuming that ovulation occurs once per year and leads to pregnancy in 82% of all cases. This latter chance follows from the ratio between the inter-ovulation interval (4.17 yrs) and the inter-birth interval (5.1 yrs), as reported by [3]. Time constant *T_R_* modulates the increase in resource assimilation with (calf) age and represents the age at which feeding efficiency is 50%. No empirical estimates are available for *T_R_* and by default we assume *T_R_* = 500 days.

## Mortality rates

Bloch *et al.* [2] calculate age-specific survival estimates for females from the age-distribution of 1,482 North-East Atlantic long-finned pilot whales. We transform these age-specific survival estimates (*P_a_*) to yearly mortality rates *z_a_* = – ln(*P_a_*) / 365 and use these to fit non-linear least squares estimates for the daily mortality rate function in Table 1. According to [2], post-weaning males experience a more continuous decline in age class frequency compared to females, and post-weaning male mortality ($\mu_{male}$) was estimated at 0.0788 year^-1^ = 2.13·10^-4^ day^‑1^. We assume that male calves experience the same mortality rate as female calves and upon weaning, the mortality for males changes to the age-independent value of $\mu_{male}$ = 2.13·10^-4^ day^‑1^. The age-dependent mortality rates and their effect on survival are shown in Figure 2 of S2: Supplemental results. The starvation mortality scalar $\mu_{s}$ affects the increase in mortality rate with decreasing body condition and we adopt $\mu_{s}$ = 0.2 by default. In combination with the starvation mortality threshold of $\rho_{s}=0.15$, this leads to a starvation mortality rate of 0.1 day^-1^ for an individual with a body condition of F/W = 0.1.

*Resource parameters*

The mean annual prey productivity *K* was set to 0.15 MJ m^-3^ day^-1^. The productivity level merely scales the size of the whale population and varying this parameter does not affect the qualitative effect of disturbance on population density or individual life history. Parameter *A* (0 – 1) controls the amplitude of yearly fluctuation in prey productivity relative to the annual mean productivity *K* and was set to zero or 0.25 to resemble either an environment of constant prey productivity or an environment with seasonally changing prey productivity. Prey turn-over rate $\delta$ controls the mean retention time of available prey in the system and was set to 0.05, which equals a mean retention time of 20 days. The volume scalar *V* = 1e6 allowed for a conversion between the volumetric density of whales and prey. The value of 𝑉 was chosen for numerical convenience and did not qualitatively affect model dynamics.

## Non-linearity parameters

Parameters that control the shape of various functions are $\eta$, $\gamma$, $\xi_{m}$, and $\xi_{c}$. No empirical estimates are available for these parameters and their default values were mainly based on qualitative reasoning. In addition, we present a sensitivity analysis for these parameters together with parameters *T_N_*, *T_R_* and $\mu_{s}$ in S2 Supplemental results. The effect of changing the values of these parameters on their constituent functions can be seen in Figure 3 of S2 Supplemental results.

Parameter $\eta$ controls the steepness of the response of milk and resource assimilation to body condition (Table 2) and its default value was set to 15. Parameter $\gamma$ determines the non-linearity of the dependency of resource feeding efficiency on age. The default value of 2.0 leads to a decelerating increase in feeding efficiency, and in combination with the default value of *T_R_* = 500, results in a feeding efficiency around 86% at weaning. Parameter $\xi_{m}$ controls the non-linearity of the response of female milk provisioning to female body condition. We assume that the decrease in milk provisioning increases at lower body conditions and therefore choose a negative default value of -2. Parameter $\xi_{c}$ controls the non-linearity of the decrease in milk feeding by the calf as a function of calf age, and the default value of $\xi_{c}=0.9$ ensures that this decrease gradually becomes stronger with increasing calf age.

# References

1. International Whaling Commission. Biology of Northern Hemisphere Pilot Whales. A collection of papers. In: Donovan GP, Lockyer CH, Martin AR, editors. Report of the International Whaling Commission, Special Issue 14. Cambridge; 1993.

2. Bloch D, Lockyer C, Zachariassen M. Age and Growth Parameters of the Long-Finned Pilot Whale off the Faroe Islands. Report of the International Whaling Commission, Special Issue 14. 1993; 163–207.

3. Martin AR, Rothery P. Reproductive Parameters of Female Long-Finned Pilot Whales (Globicephala melas) Around the Faroe Islands. Report of the International Whaling Commission, Special Issue 14. 1993; 263–304.

4. Lockyer C. Seasonal Changes in Body Fat Condition of Northeast Atlantic Pilot Whales, and their Biological Significance. Report of the International Whaling Commission, Special Issue 14. 1993; 325–350.

5. Kleiber M. The fire of life: an introduction to animal energetics. Huntington, N.Y.: R.E. Krieger Pub. Co.; 1975.

6. Brody S. Bioenergetics and growth. New York, NY: Hafner Publishing Co; 1968.

7. Illius AW, O’Connor TG. Resource heterogeneity and ungulate population dynamics. Oikos. 2000;89: 283–294. doi:10.1034/j.1600-0706.2000.890209.x

**Table 1**: Model parameters

| Symbol | Unit | Value | Description |
| --- | --- | --- | --- |
| $\boldsymbol{T}_{\boldsymbol{P}}$ | $\text{day}$ | 365 | Gestation period |
| $\boldsymbol{T}_{\boldsymbol{L}}$ | $\text{day}$ | 1223 | Lactation period (age at weaning) |
| $\boldsymbol{T}_{\boldsymbol{N}}$ | $\text{day}$ | 365 | Age at which milk consumption starts to decrease |
| $\boldsymbol{T}_{\boldsymbol{R}}$ | $\text{day}$ | 500 | Age at which prey feeding efficiency is 50% |
| $\boldsymbol{l}_{\boldsymbol{b}}$ | $\text{cm}$ | 177 | Structural length at birth |
| $\boldsymbol{l}_{\boldsymbol{\infty}}$ | $\text{cm}$ | 450 (females) | Ultimate structural length |
|  |  | 594 (males) |  |
| $\boldsymbol{k}$ | $\text{day}^{\text{–1}}$ | 0.00045 (females) | Von Bertalanffy growth rate |
|  |  | 0.00028 (males) |  |
| $\boldsymbol{\omega}_{\mathbf{1}}$ | $\text{kg·}\text{cm}^{\text{–}\omega_{2}}$ | 8.5·10^–5^ | Structural mass-length scaling constant |
| $\boldsymbol{\omega}_{\mathbf{2}}$ | – | 2.6 | Structural mass-length scaling exponent |
| $\boldsymbol{\theta}_{\boldsymbol{F}}$ | – | 0.2 | Relative metabolic cost of reserves |
| $\boldsymbol{\rho}$ | – | 0.3 | Target body condition threshold |
| $\boldsymbol{\rho}_{\boldsymbol{s}}$ | – | 0.15 | Starvation body condition threshold |
| $\boldsymbol{\phi}_{\boldsymbol{R}}$ | $\text{m}^{3}\text{·}\text{kg}^{\text{–2/3}}\text{·}\text{day}^{-1}$ | 1 or 0 | Prey encounter rate scalar. 0 during disturbance |
| $\boldsymbol{\phi}_{\boldsymbol{L}}$ | $\text{MJ·}\text{kg}^{\text{–2/3}}\text{·}\text{day}^{\text{–1}}$ | 2.7 | Lactation scalar |
| $\boldsymbol{\eta}$ | – | 15 | Steepness of assimilation response around target body condition |
| $\boldsymbol{\gamma}$ | – | 2 | Shape parameter of prey assimilation-age response |
| $\boldsymbol{\xi}_{\boldsymbol{m}}$ | – | –2 | Non-linearity in female body condition-milk provisioning relation |
| $\boldsymbol{\xi}_{\boldsymbol{c}}$ | – | 0.9 | Non-linearity in milk assimilation-calve age relation |
| $\boldsymbol{\sigma}_{\boldsymbol{M}}$ | $\text{MJ·}\text{kg}^{\text{–3/4}}\text{∙}\text{day}^{\text{–1}}$ | 0.75 | Field metabolic rate scalar |
| $\boldsymbol{\sigma}_{\boldsymbol{G}}$ | $\text{MJ·}\text{kg}^{\text{–1}}$ | 30 | Energetic costs per unit structural mass growth |
| $\boldsymbol{\sigma}_{\boldsymbol{L}}$ | – | 0.86 | Lactation conversion efficiency |
| $\boldsymbol{\alpha}_{\mathbf{1}}$ | $\text{day}^{\text{–1}}$ | 4.01·10^–4^ | Mortality parameter females & calves |
| $\boldsymbol{\beta}_{\mathbf{1}}$ | $\text{day}^{\text{–1}}$ | 5.82·10^–4^ | Mortality parameter females & calves |
| $\boldsymbol{\alpha}_{\mathbf{2}}$ | $\text{day}^{\text{–1}}$ | 6.04·10^–6^ | Mortality parameter females & calves |
| $\boldsymbol{\beta}_{\mathbf{2}}$ | $\text{day}^{\text{–1}}$ | 3.01·10^–4^ | Mortality parameter females & calves |
| $\boldsymbol{\mu}_{\boldsymbol{male}}$ | $\text{day}^{\text{–1}}$ | 2.13·10^-4^ | Mortality parameter males |
| $\boldsymbol{\mu}_{\boldsymbol{s}}$ | $\text{day}^{\text{–1}}$ | 0.2 | Starvation mortality scalar |
| $\boldsymbol{\psi}$ | $\text{day}^{\text{–1}}$ | 0.00225 | Daily chance of becoming pregnant |
| $\boldsymbol{\varepsilon}_{\boldsymbol{+}}$ | $\text{MJ·}\text{kg}^{\text{–1}}$ | 55 | Anabolic reserves conversion efficiency |
| $\boldsymbol{\varepsilon}_{\boldsymbol{-}}$ | $\text{MJ·}\text{kg}^{\text{–1}}$ | 35 | Catabolic reserves conversion efficiency |
| $\boldsymbol{\delta}$ | $\text{day}^{\text{–1}}$ | 0.05 | Prey turn-over rate |
| $\boldsymbol{K}$ | $\text{MJ∙}\text{m}^{-3}\text{∙}\text{day}^{\text{–1}}$ | 0.15 | Mean annual prey productivity |
| $\boldsymbol{A}$ | – | 0 or 0.25 | Relative amplitude of seasonal fluctuation in prey productivity |
| $\boldsymbol{V}$ | – | 1·10^6^ | Volume scalar between whale and prey density |
| $\boldsymbol{t}_{\boldsymbol{start}}$ | $\text{day}$ | 0 or 182.5 | Starting day of disturbance event |
| $\boldsymbol{t}_{\boldsymbol{dist}}$ | $\text{day}$ | 0 – 50 | Duration of disturbance event |

**Table 2**: Model equations with description and units

| **State-variables** | |  |  | |
| --- | --- | --- | --- | --- |
| Time | | $t$ | $\text{day}$ | |
| Prey density | | $R$ | $\text{MJ ∙ }\text{m}^{-3}$ | |
| Age | | $a$ | $\text{day}$ | |
| Time since conception | | $\tau_{p}$ | $\text{day}$ | |
| Reserve mass | | $F$ | $\text{kg}$ | |
| Structural length foetus | | $l_{p}\left( \tau_{p} \right)=l_{b}\frac{\tau_{p}}{T_{P}}$ $0\leq\tau_{p}\leq T_{P}$ | $\text{cm}$ | |
| Structural length non-foetus | | $l\left( a \right)=l_{\infty}-\left( l_{\infty}-l_{b} \right)e^{-ka}$ | $\text{cm}$ | |
| Structural mass | | $S\left( l \right)=\omega_{1}l^{\omega_{2}}$ | $\text{kg}$ | |
| Total mass | | $W\left( S,F,\tau_{p} \right)=\left\{ \begin{matrix} S+F+S\left( l_{p}\left( \tau_{p} \right) \right) & \text{pregnant} \\ S+F & \text{otherwise} \end{matrix} \right.$ | $\text{kg}$ | |
| Maintenance mass | | $W_{M}\left( S,F,\tau_{p} \right)=\left\{ \begin{matrix} S+\theta_{F}F+S\left( l_{p}\left( \tau_{p} \right) \right) & \text{pregnant} \\ S+\theta_{F}F & \text{otherwise} \end{matrix} \right.$ | $\text{kg}$ | |
| **Energetic rates** | |  |  | |
| Prey assimilation | | $I_{R}\left( R,a,S,F,W \right)=\phi_{R}RS^{2/3}\frac{a^{\gamma}}{T_{R}^{\gamma}{+ a}^{\gamma}} \frac{1}{1+e^{-\eta(\rho W/F-1)}}$ | $\text{MJ ∙ }\text{day}^{-1}$ | |
| Milk assimilation | | $\begin{matrix} I_{L}\left( a,S,F,W,F_{m},W_{m} \right)=\phi_{L}S^{2/3}\frac{1}{1+e^{-\eta(\rho W/F-1)}}\times\\ \text{min}\left( 1,\left[ \frac{1-\frac{a-T_{N}}{T_{L}-T_{N}}}{1-\xi_{c}\frac{a-T_{N}}{T_{L}-T_{N}}} \right]_{+} \right)\left[ \frac{\left( 1-\xi_{m} \right)\left( F_{m}-\rho_{s}W_{m} \right)}{\left( \rho-\rho_{s} \right)W_{m}-\xi_{m}\left( F_{m}-\rho_{s}W_{m} \right)} \right]_{+} \end{matrix}$ | $\text{MJ ∙ }\text{day}^{-1}$ | |
| Field metabolic rate | | $C_{M}\left( W_{M} \right)=\sigma_{M}W_{M}^{3/4}$ | $\text{MJ ∙ }\text{day}^{-1}$ | |
| Structural mass growth | | ${C_{G}\left( l \right)=\sigma}_{G}\omega_{1}k\left( l_{\infty}-l \right)\omega_{2}l^{\omega_{2}-1}$ | $\text{MJ ∙ }\text{day}^{-1}$ | |
| Fetal development | | ${C_{P}\left( \tau_{p} \right)=\sigma}_{G}\omega_{1}\omega_{2}\left( \frac{l_{b}}{T_{P}} \right)^{\omega_{2}}{\tau_{p}}^{\omega_{2}-1}$ $0\leq\tau_{p}\leq T_{P}$ | $\text{MJ ∙ }\text{day}^{-1}$ | |
| Lactation costs | | ${C_{L}\left( F,W,a_{c},S_{c},F_{c},W_{c} \right)=I}_{L}\left( a_{c},S_{c},F_{c},W_{c},F,W \right)/\sigma_{L}$ | $\text{MJ ∙ }\text{day}^{-1}$ | |
| **Mortality** | |  |  | |
| Female and calf | | ${D_{a}\left( a \right)=\alpha}_{1}e^{{-\beta}_{1}a}+\alpha_{2}e^{\beta_{2}a}$ | $\text{day}^{-1}$ | |
| Non-calf males | | $D_{a}=\mu_{male}$ | $\text{day}^{-1}$ | |
| Starvation | | $D_{s}\left( F,W \right)=\mu_{s}\left( \frac{\rho_{s}W}{F}-1 \right)\text{,}$ $F<\rho_{s}W$ | $\text{day}^{-1}$ | |
| **Other** | |  |  | |
| Reserve mass needed  for one neonate | | $F_{neo}=\frac{\sigma_{G}\omega_{1}{l_{b}}^{\omega_{2}}}{\varepsilon{}_{-}}+\frac{\rho_{s}\omega_{1}{l_{b}}^{\omega_{2}}}{(1-\rho_{s})}$ | $\text{kg}$ | |
| Pregnancy threshold | | $F=\rho_{s}W+F_{neo}$ | $\text{kg}$ | |
| Prey dynamics | | $\frac{dR}{dt}=K\left( 1.0-A\cos\left( \frac{2\pi t}{365} \right) \right)-\delta R-V^{-1}\sum_{i} I_{R}\left( R,a_{i},S_{i},F_{i},W_{i} \right)$ | $\text{MJ ∙ }\text{m}^{-3}\text{ ∙ }\text{ day}^{-1}$ | |
| Disturbance | $\phi_{R}=\left\{ \begin{matrix} 0 & \text{if} \left( \left( t \text{mod }365 \right)\geq t_{start} \right) \& \left( \left( t \text{mod} 365 \right)<\left( t_{start}+t_{dist} \right) \right) \\ 1 & \text{otherwise} \end{matrix} \right.$ | | |  |
| A ‘+’-subscript indicates that only positive values are used, *i.e.* $\left[ f(x) \right]_{+}=\max(f(x), 0)$ | | | | |
