# Supplementary material for: Density dependence can obscure nonlethal effects of disturbance on life history of medium-sized cetaceans
Source: PLoS One. 2021 Jun 3;16(6):e0252677. doi: 10.1371/journal.pone.0252677 (PMC8174747; doi:10.1371/journal.pone.0252677)
Supplement: S2 File — (PDF) [file pone.0252677.s002.pdf]

S2 File. Supplemental results

Supplement to:

"*Density dependence can obscure nonlethal effects of disturbance on life history of medium-sized cetaceans*"

Submitted to PlosONE

Seasonal prey productivity

Seasonal variation in prey productivity did not change the qualitative effect of disturbance on whale and prey density, although it resulted in a reduced mean whale density in the absence of disturbance (Fig. 5). Compared to constant prey productivity, the disturbance duration at which the whale population went extinct was 2 days longer with seasonality and summer disturbance, and 6 days shorter with seasonality and winter disturbance. Variation in prey density was lowest in non-seasonal environments. In seasonal environments, disturbance in summer caused larger variation in prey density than disturbance in winter.

The improvement in body condition with disturbance also occurred in seasonal environments (Fig. 6). In contrast to a constant prey productivity, seasonality led to a bimodal distribution of body condition for most reproductive classes, with the high and low peaks corresponding to the body conditions during 'summer' and 'winter', respectively.

The effect of disturbance on age-specific patterns of reproduction and survival were modified by seasonality in prey productivity. In seasonal environments reproduction started earlier with increasing disturbance, similar to effect of disturbance with constant productivity. However, seasonality ($A = 0.25$) evened out the effect of disturbance on reproductive output when disturbance occurred in winter, and reversed this effect when disturbance occurred in summer (Fig. 7). With summer disturbance in seasonal environments, age-specific reproductive output was therefore highest in undisturbed populations and lowest with 30 days of disturbance for most ages except for the first few reproducing years. This exception resulted from the earlier onset of reproduction with increasing disturbance, which also occurred in seasonal environments.

Compared to a constant environment, seasonality in productivity led to a strong density-dependent decrease in survival during the first years of life with no and ten days of disturbance per year (Fig. 7). With 30 days of disturbance this density-dependent effect of seasonality was relaxed, which led to increased survival during the first six to eight years of life, depending on whether disturbance happened in summer or winter. Young mature females experienced starvation-induced mortality during lactating of their first calf with 30 days of disturbance in seasonal environments and the resulting drop of survival nullified the survival advantage that was present at younger ages. Ten days of disturbance per year did not change the age-specific survival pattern compared to no

disturbance. The pattern of age-specific expected reproductive output was not affected by seasonal variation in prey productivity. With seasonal variation in prey productivity, there was a small effect of disturbance on mean life expectancy and mean reproductive output of females older than age 10 yrs (Fig. 8). With summer disturbance, mean life expectancy increased from 10.6 to 11.5 yrs between no and 40 days of disturbance and mean reproductive output of females beyond age 10 yrs slightly decreased (from 3.14 to 3.07). When disturbance happened in winter there was no noticeable effect of increased disturbance duration on these life history statistics.

Without disturbance, females living in seasonal environments had a lower AfR and a higher AfW than females living in non-seasonal environments (Fig. S8). This response to seasonality can be explained by the lower calf survival associated with seasonality. As for constant prey productivity, there was an overall downward trend in length at first reproduction, AfR and AfW with increasing disturbance duration in seasonal environments (Fig. 8).

Supplemental Figures

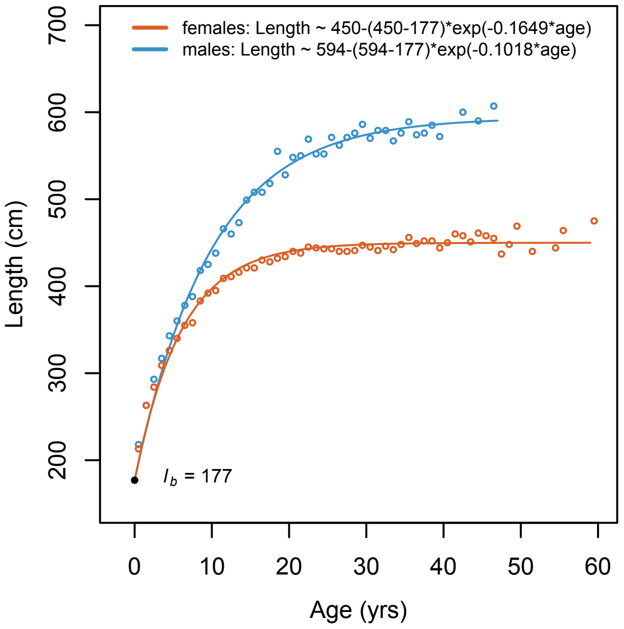

Figure 1: Length-age relationships for female and male North Atlantic long-finned pilot whales (*Globicephala melas*). Points show mean length at age data and lines show the fitted Von Bertalanffy length-age relationships, which was fixed at $l_b = 177$.

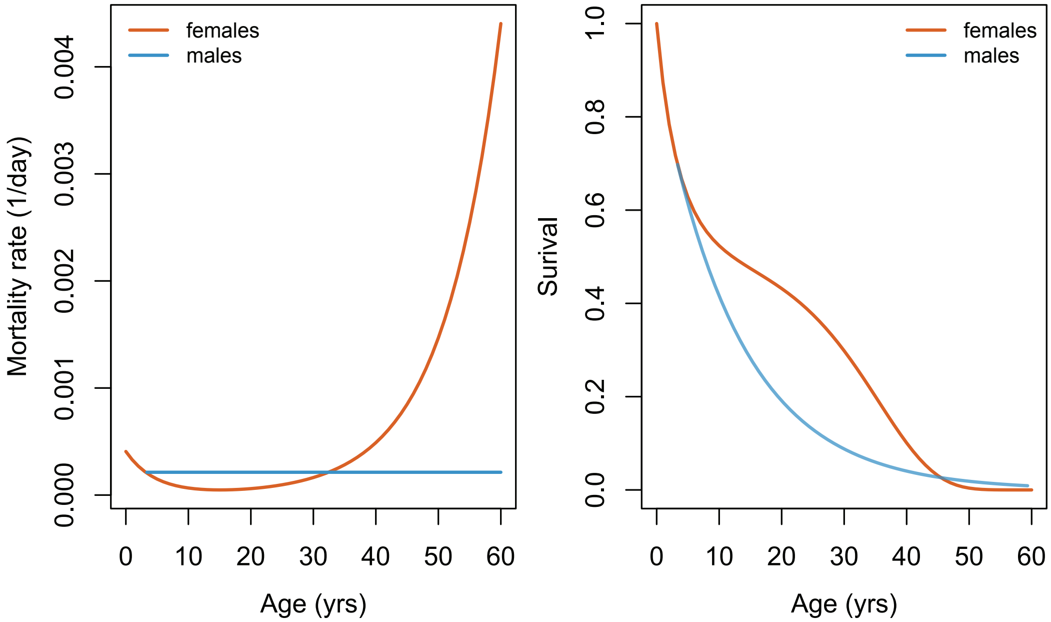

Figure 2: Age-dependent mortality rates (left) and age-dependent cumulative survival for males and females, in absence of starvation mortality. Mortality rates of male calves is identical to that of female calves.

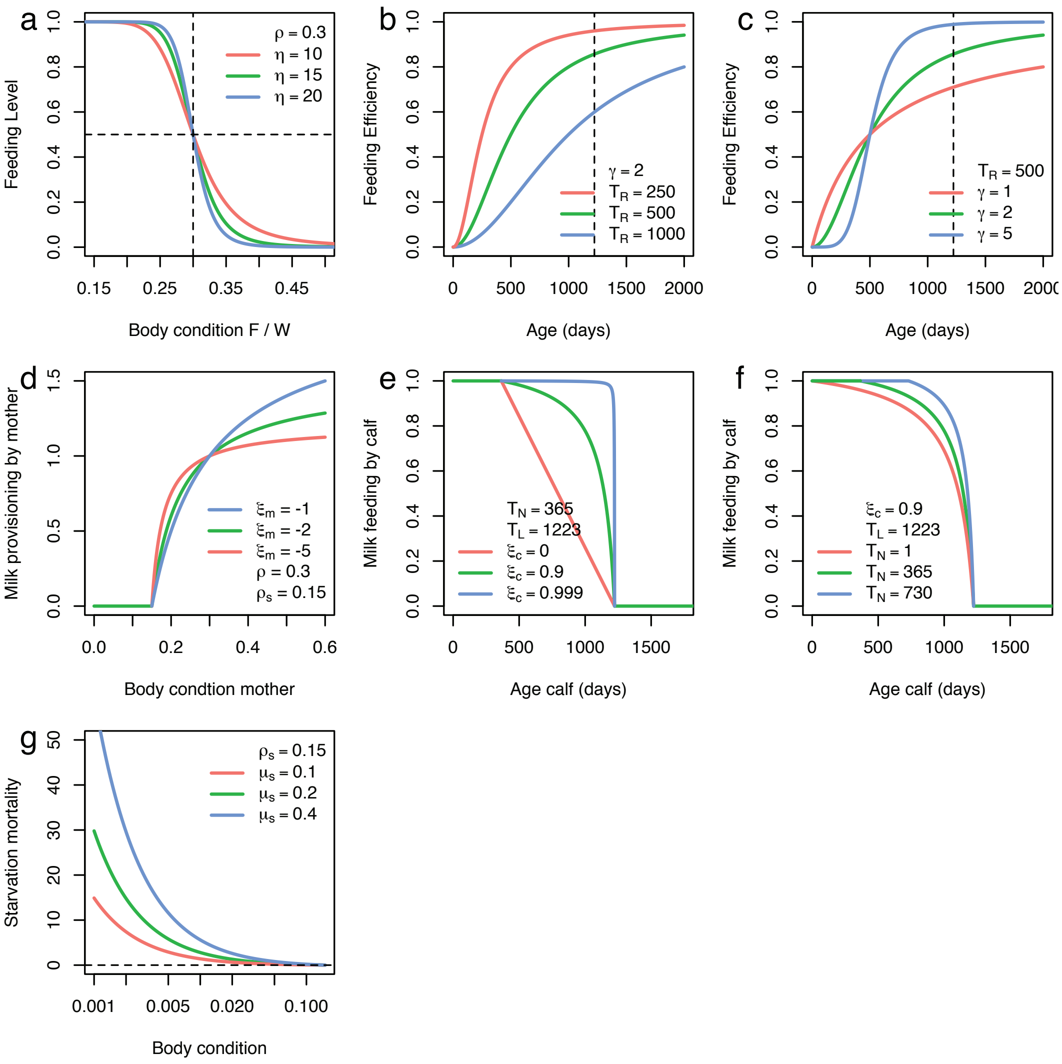

Figure 3: Functions related to prey feeding, lactation and starvation mortality and the effect of changes in parameters on the shape of these functions. In a), the feeding level $\left(\dfrac{1}{1+e^{-\eta(\rho W/F-1)}}\right)$ determines both prey and milk assimilation rate and is plotted as a function of body condition (F/W). In b) and c), the feeding efficiency $\left(\dfrac{a^\gamma}{T_R^\gamma + a^\gamma}\right)$ affects resource assimilation rate. In d), milk provisioning represents the component $\left(\left[\dfrac{(1-\xi_m)(F_m-\rho_s W_m)}{(\rho-\rho_s)W_m-\xi_m(F_m-\rho_s W_m)}\right]_+\right)$ of the lactation rate function and related to the female (subscript m). Panels e) and f) show the dependency of lactation on calf age $\left(\min\left(1, \left[\dfrac{1-\frac{a-T_F}{T_L-T_F}}{1-\xi_c\frac{a-T_F}{T_L-T_F}}\right]_+\right)\right)$. Lastly, panel g) shows the starvation mortality rate as a function of body condition. For each panel, default parameter values are plotted in green.

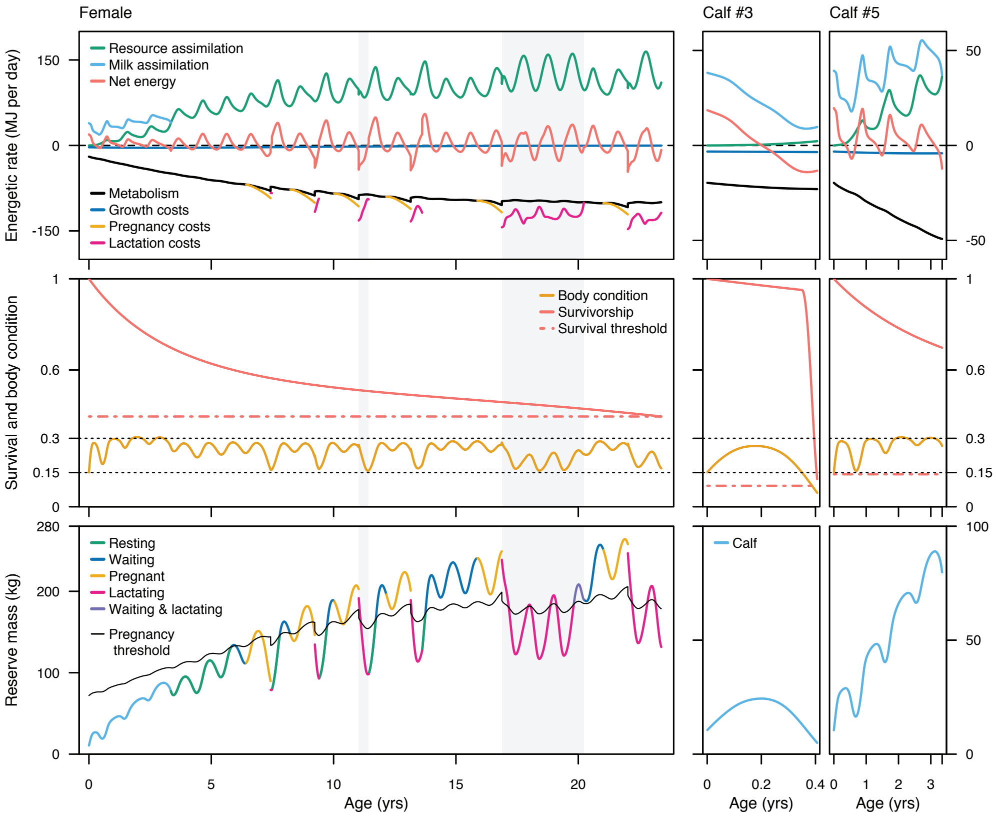

Figure 4: Energetic rates (top panels), survival and body condition (middle panels) and reserve mass (bottom panels) of a female and two of her calves (calf #3 and calf #5, indicated with grey shadings in female panels). Calf #3 died at age of 148 days following a rapid decrease in survival due to starvation mortality when body condition dropped below the starvation threshold ($\rho_s = 0.15$; lower dotted line in middle panels). Calf #5 survived until weaning ($a = 1223$ days ≈ 3.35 yrs.) and eventually became 37.8 yrs. old. In top panels, milk assimilation is plotted on top of prey assimilation. Similarly, pregnancy and lactation costs are plotted on top of metabolic and growth costs, and metabolic costs are plotted on top of growth costs. Net energy represents the sum of all energetic rates and indicates whether reserve mass increases or decreases (positive or negative values of net energy). Each individual died when its survivorship reached the survival threshold (shown in middle panel). In middle panels, the top dotted line indicates the target body condition threshold $\rho = 0.30$. In bottom panels, reserve mass is colored according to reproductive status. Note that the female entered the waiting period during the end of the lactation period of calf #5. Prey productivity is seasonal ($A = 0.25$) and other parameters at default values (Table 1 of S1: Model parameterization).

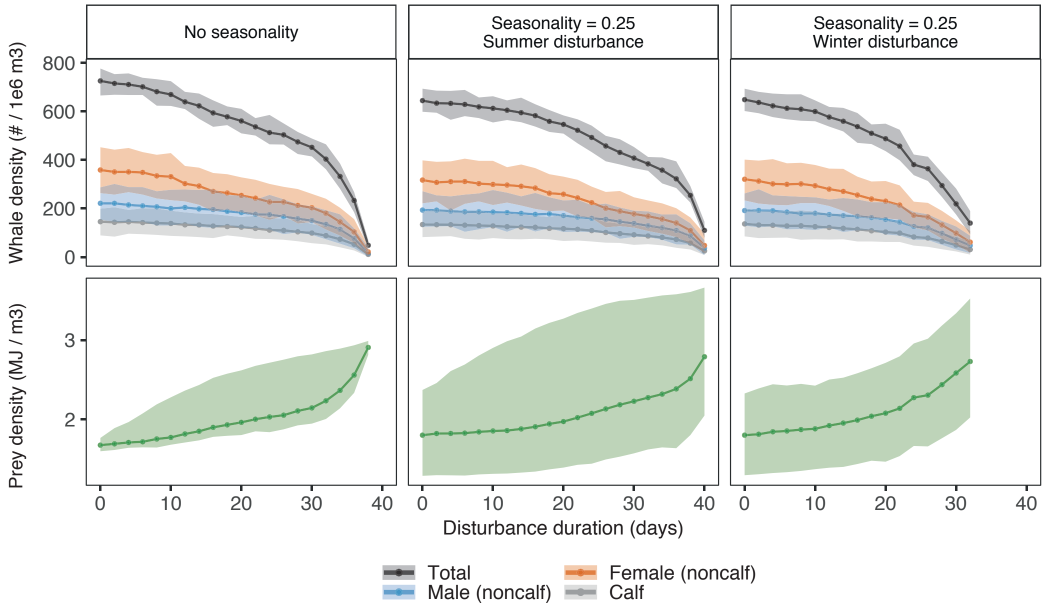

Figure 5: Population density (top panels) and prey density (bottom panels) as a function of disturbance duration (t_{dist}). In environments with seasonal prey productivity ($A = 0.25$), summer disturbance ($t_{start} = 182.5$) happened in the season of high prey productivity, while winter disturbance ($t_{start} = 182.5$) happened during low productivity. Each point represents the mean density of a simulation of 200 years, after an initial 200-year period to allow for population transients. Output was collected every 5 days (14600 observation per point). Lines connect mean densities and shaded areas indicate min/max density.

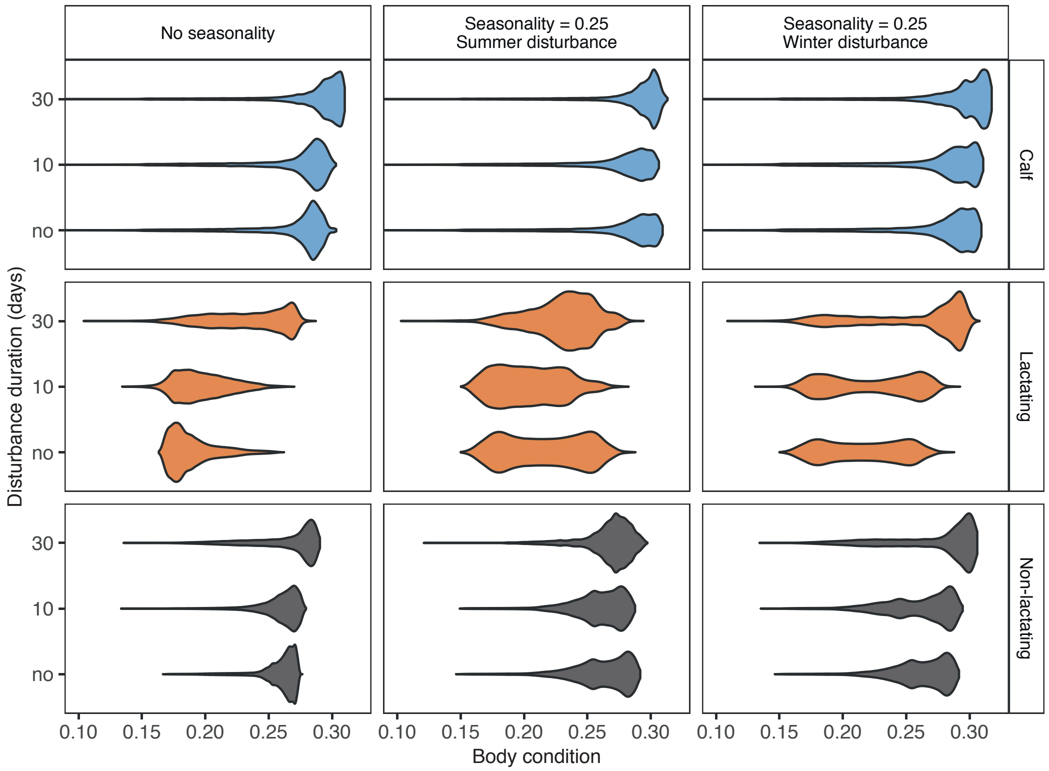

Figure 6: Distributions of body condition per reproductive class for different disturbance durations (t_{dist}). Calves ($a < 1223$ days) are plotted as a separate reproductive class. Females that are simultaneously lactating and waiting or lactating and pregnant are referred to as lactating. Females that were resting, waiting or pregnant are collectively referred to as non-lactating. Data in each panel is derived from a single simulation of 100 years with data collected every 10 days for 1000 different females. Different environment scenarios as in Fig. 5.

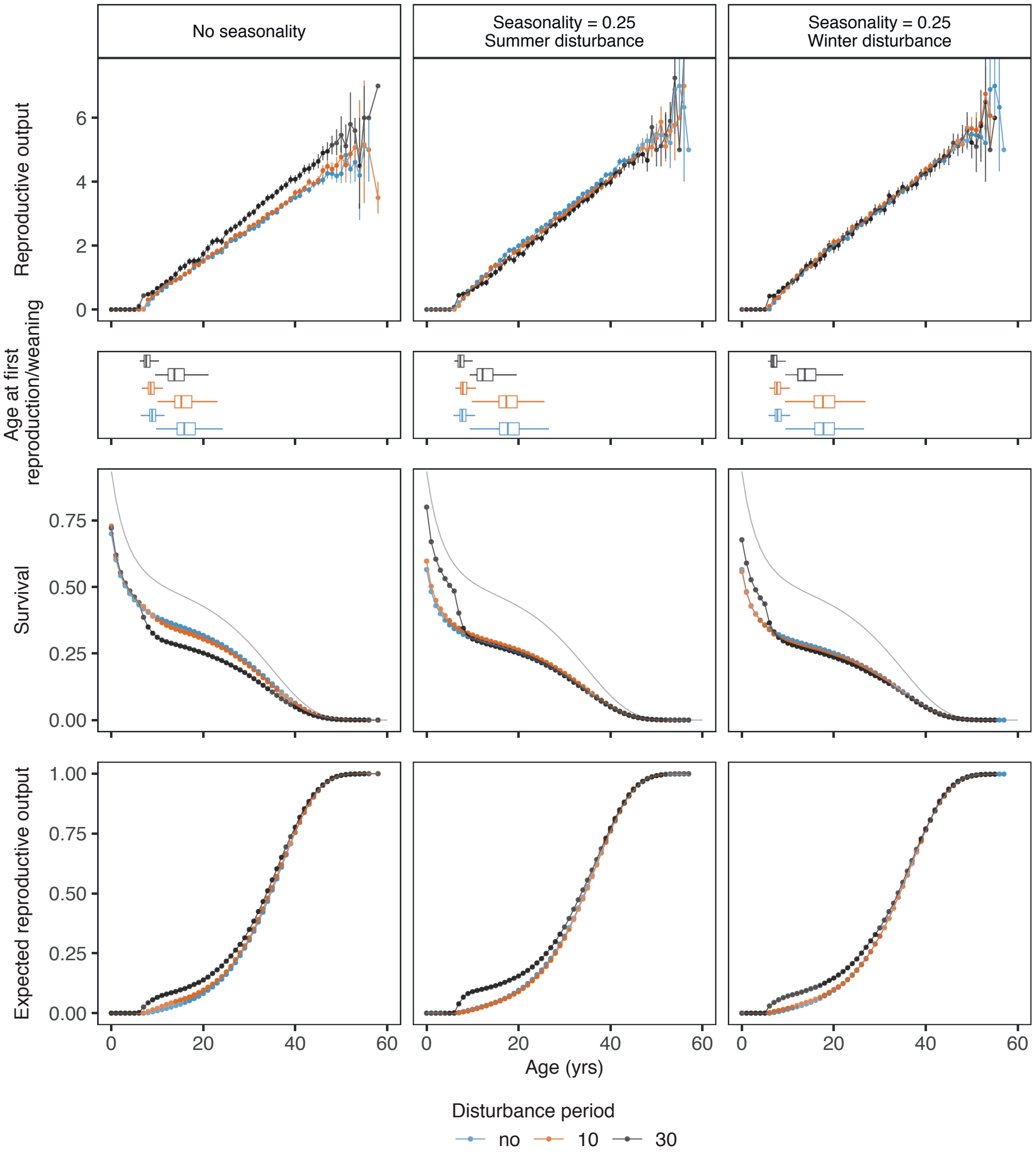

Figure 7: Age-specific patterns of survival and reproduction for different disturbance durations (colors) and environmental scenarios. Plotted variables (rows) as in main text Fig. 4 and different environment scenarios (columns) as in Fig. 5

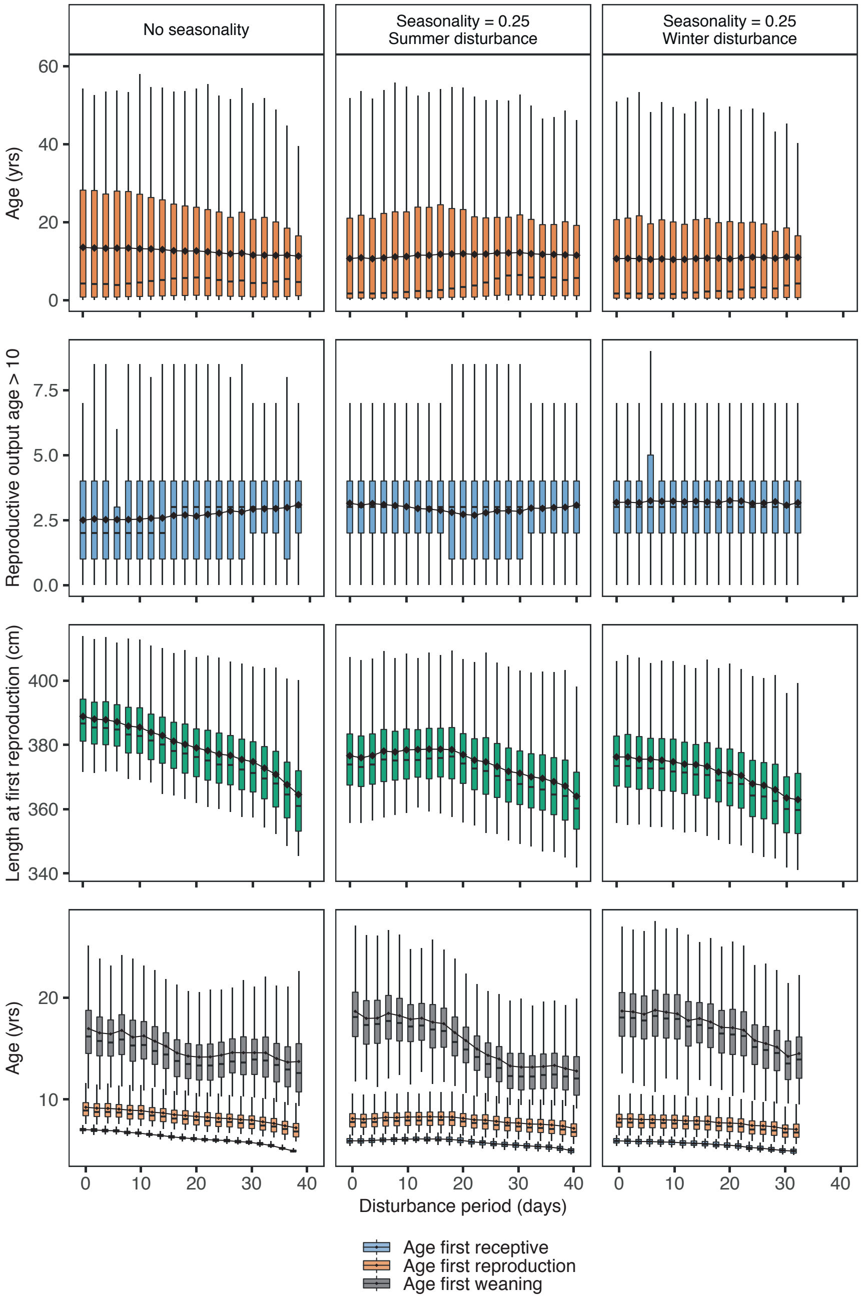

Figure 8: Distribution of life expectancy (top panels), lifetime reproductive output for females older than 10 years (second row), female length at first reproduction (third row) and female age at first receptive, first reproduction and weaning of first calf (bottom panels) as a function of disturbance duration. Different environment scenarios (columns) as in Fig. 5. All symbols as in main text Fig. 5. The legend applies to bottom panel only.

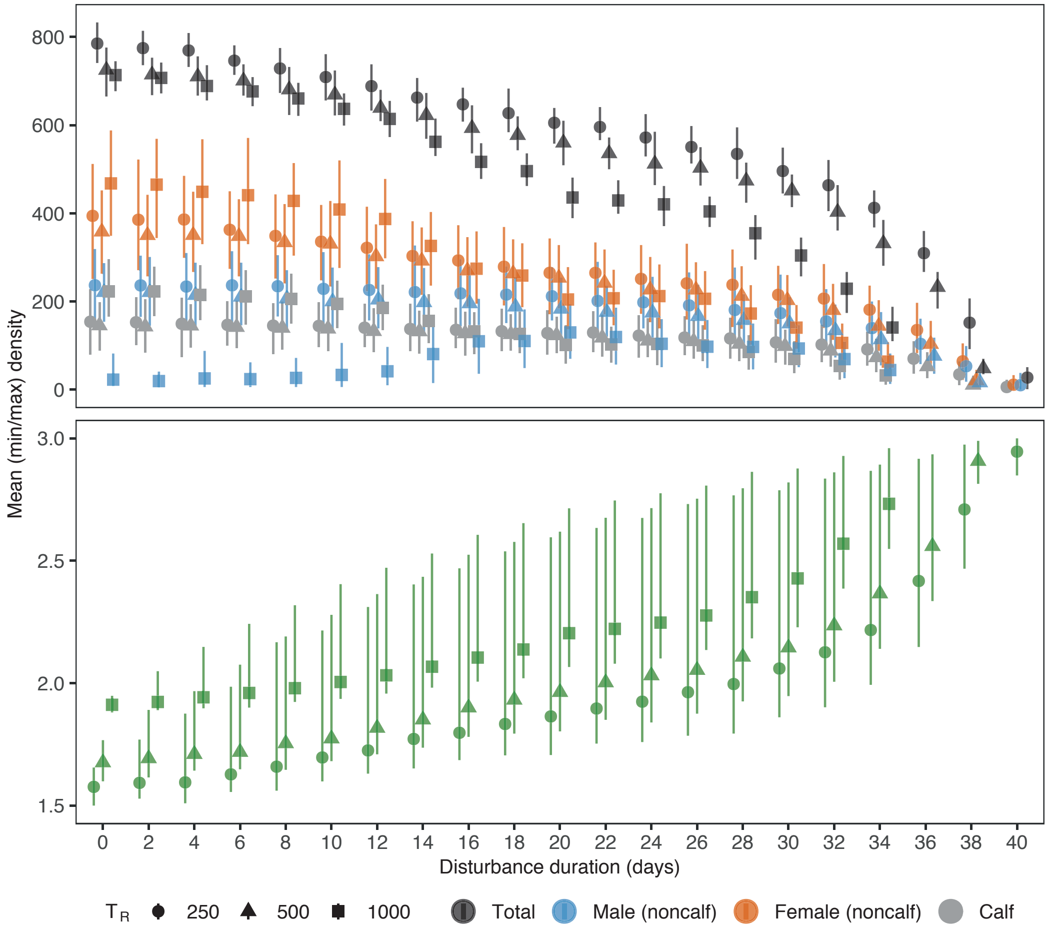

Figure 9: Sensitivity of whale and prey density to parameter T_R, which modifies the increase in resource feeding efficiency with age and sets the age at which resource feeding efficiency is 50%. Default value is $T_R = 500$ (triangles), for which feeding efficiency at weaning ($a = 1223$) is 86%. Population response to disturbance is altered at $T_R = 1000$, for which feeding efficiency at weaning is 60%. This led to starvation mortality among young males and changed the sex ratio towards more female-dominated.

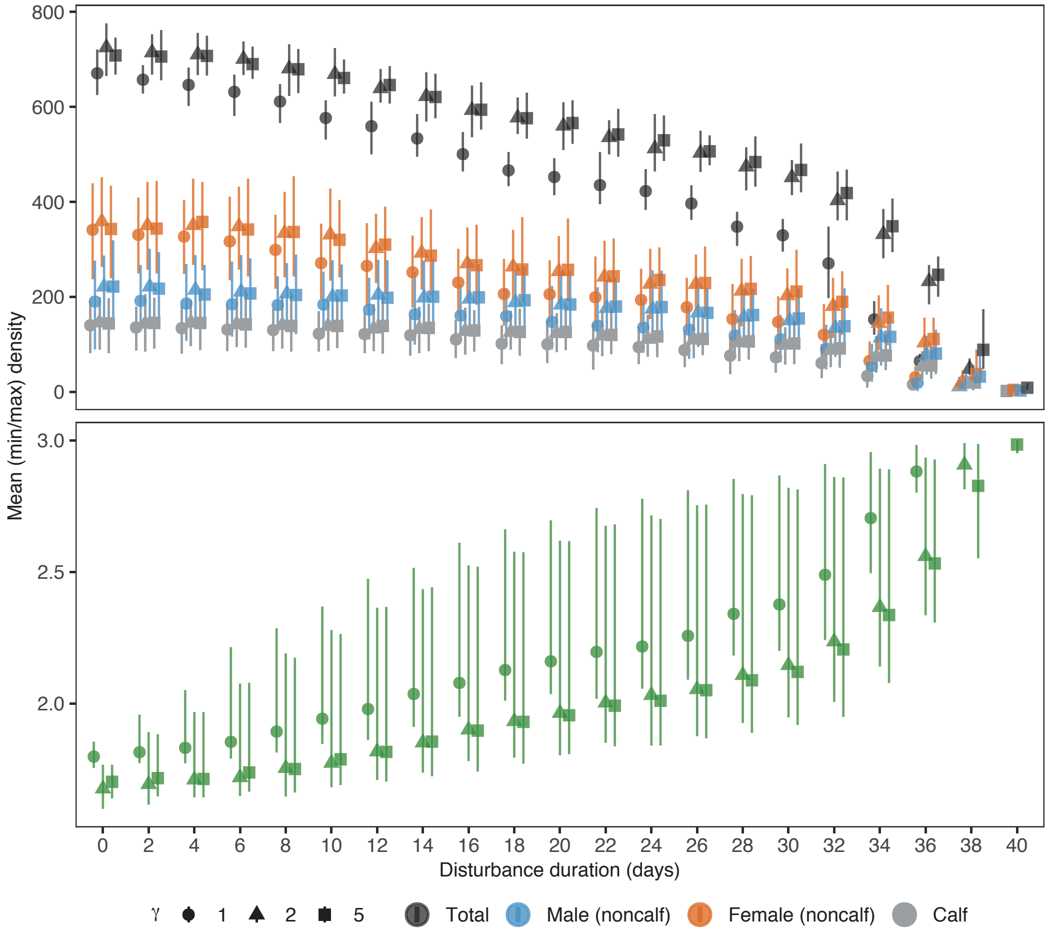

Figure 10: Sensitivity of whale and prey density to parameter γ, which modified the non-linearity in the increase in resource feeding efficiency with age. Default value is $\gamma = 2$ (triangles), for which feeding efficiency at weaning ($a = 1223$) is 86%. Population response to disturbance is altered at $\gamma = 1$, for which feeding efficiency at weaning is 70%.

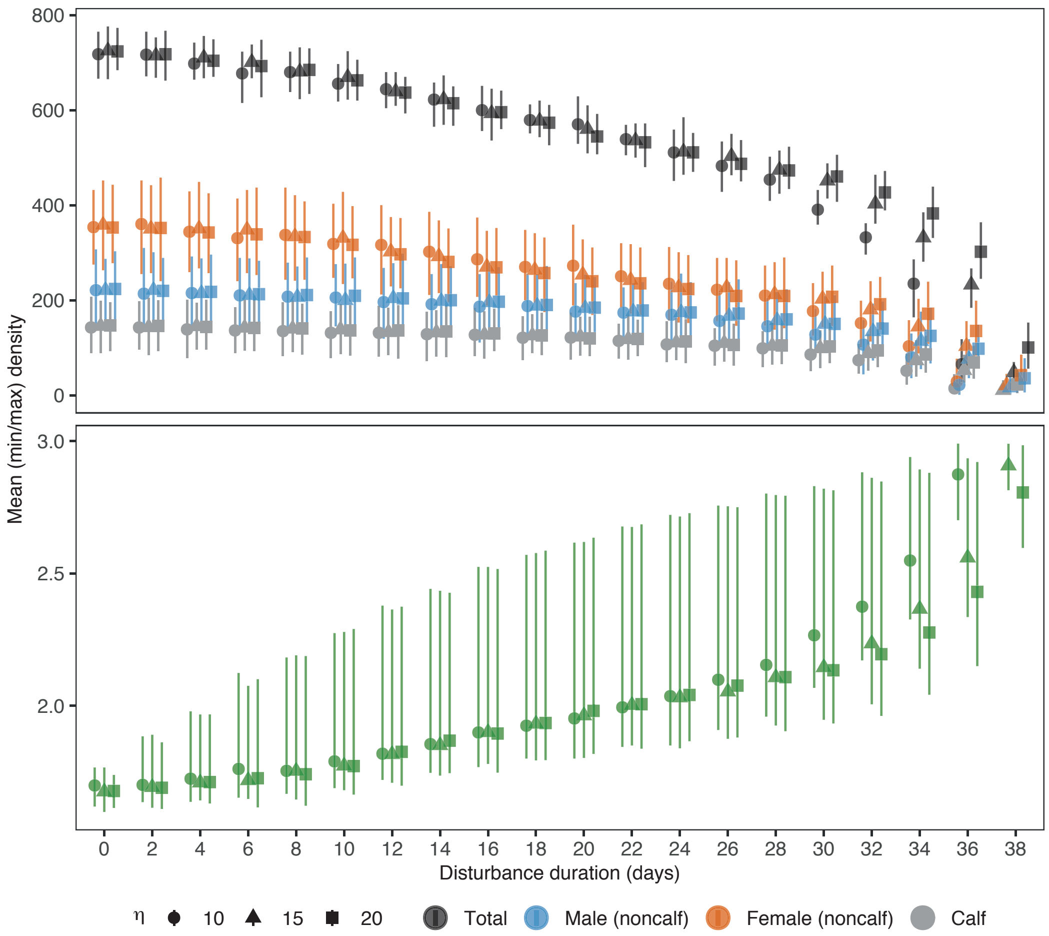

Figure 11: Sensitivity of whale and prey density to parameter η, which determined the steepness of the decrease in feeding level with increasing body condition. Default value is $\eta = 15$ (triangles) and a large value of η led to a steeper decline of feeding level with increasing body condition.

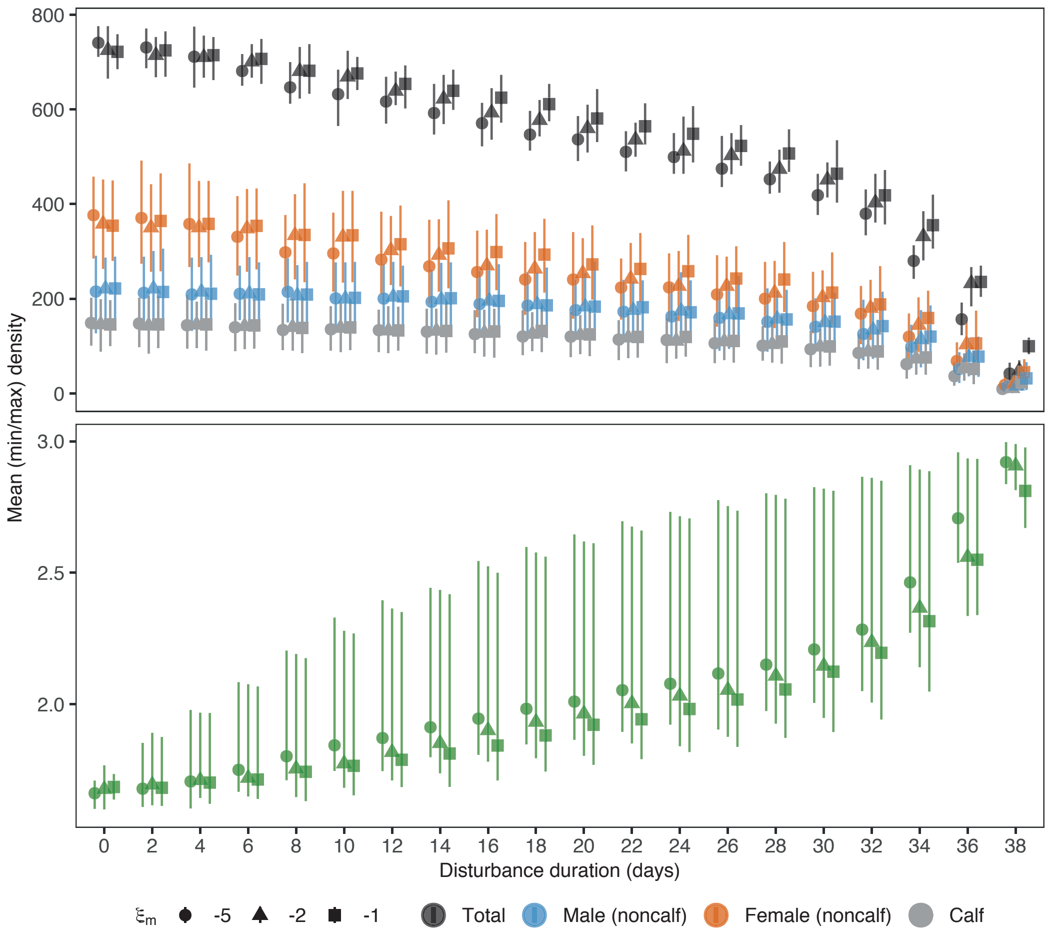

Figure 12: Sensitivity of whale and prey density to parameter ξ_m, which modified the non-linearity of rate of milk provisioning with body condition of the mother. Default value is $\xi_m = -2$ (triangles) and a lower value led to a more rapid decline of milk provisioning with decreasing female body condition.

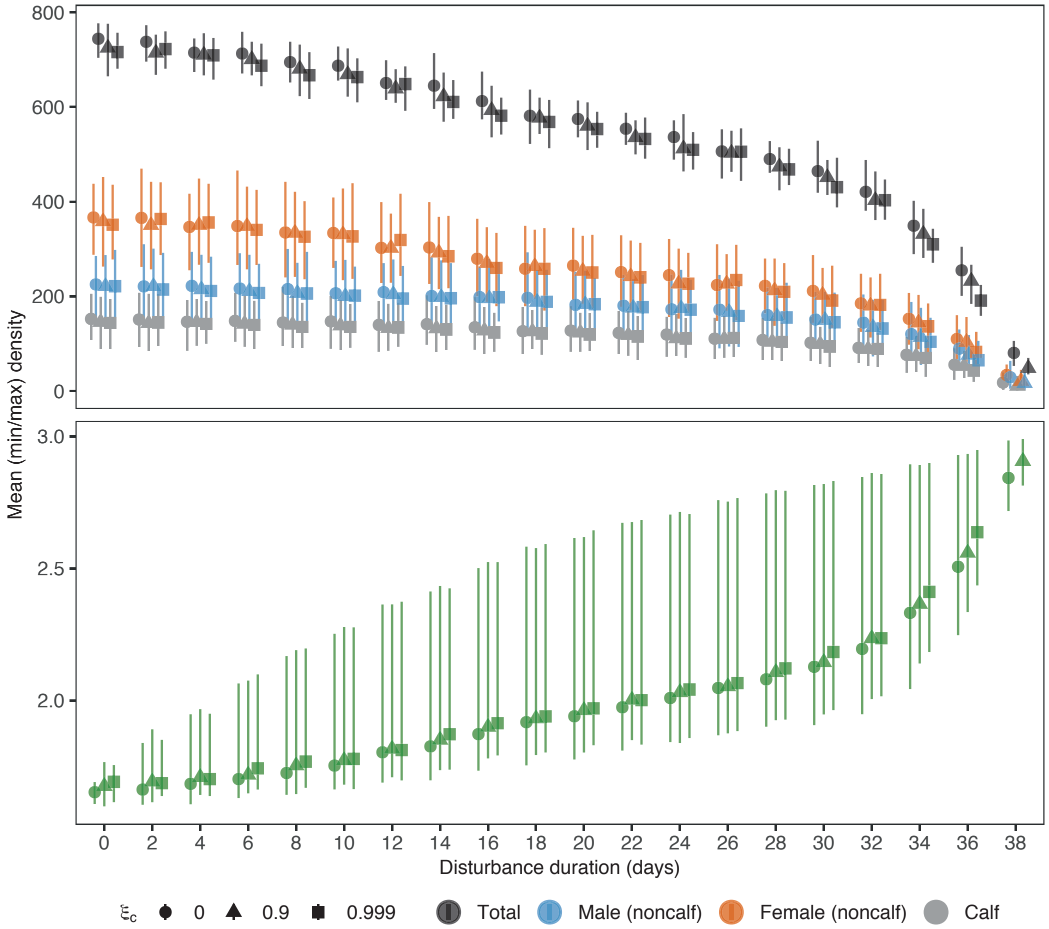

Figure 13: Sensitivity of whale and prey density to parameter ξ_c, which modified the shape of the decline in milk feeding efficiency by the calf with calf age. Default value is $\xi_c = 0.9$ (triangles). For $\xi_c = 0.0$, milk feeding declined linearly with calf age, and for $\xi_c = 0.999$ milk feeding was unaffected by calf age up to the age at weaning when it rapidly declined.

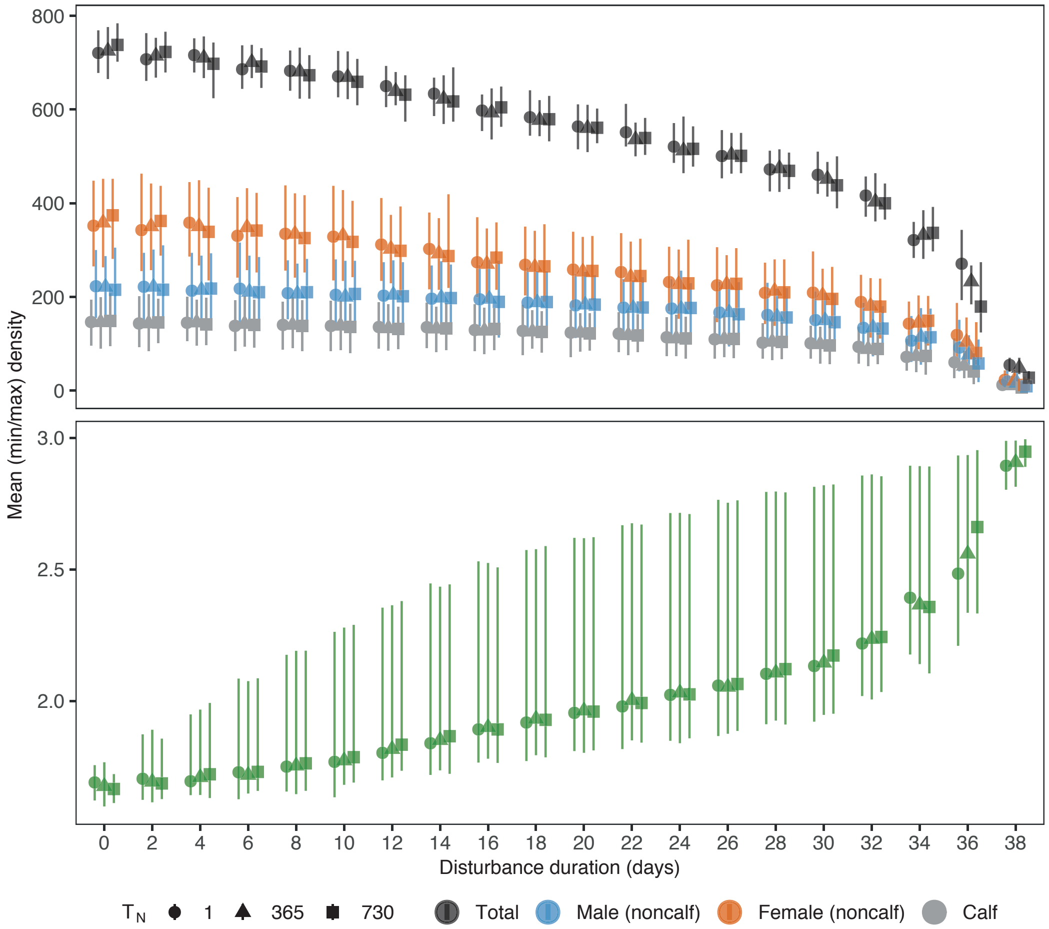

Figure 14: Sensitivity of whale and prey density to parameter T_N, which sets the age beyond which the milk feeding efficiency starts to decline with calf age. Default value is $T_N = 365$ (triangles). For $T_N = 1$, milk feeding starts to decline with calf age when the calf is one day old. For $T_N = 730$ this only happens when the calf is two years old.

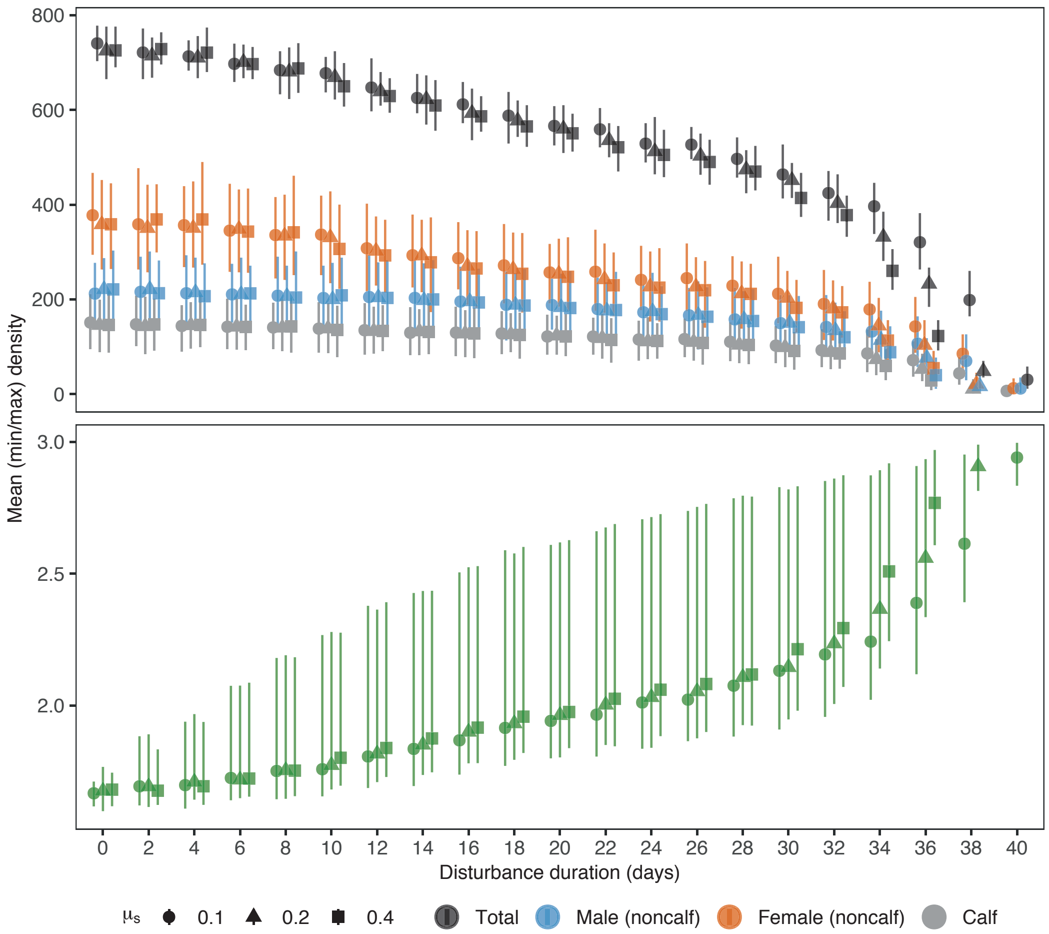

Figure 15: Sensitivity of whale and prey density to parameter μ_s, which modifies the increase of the starvation mortality rate when body condition declines below $\rho_s = 0.15$. Default value is $\mu_s = 0.2$ (triangles).

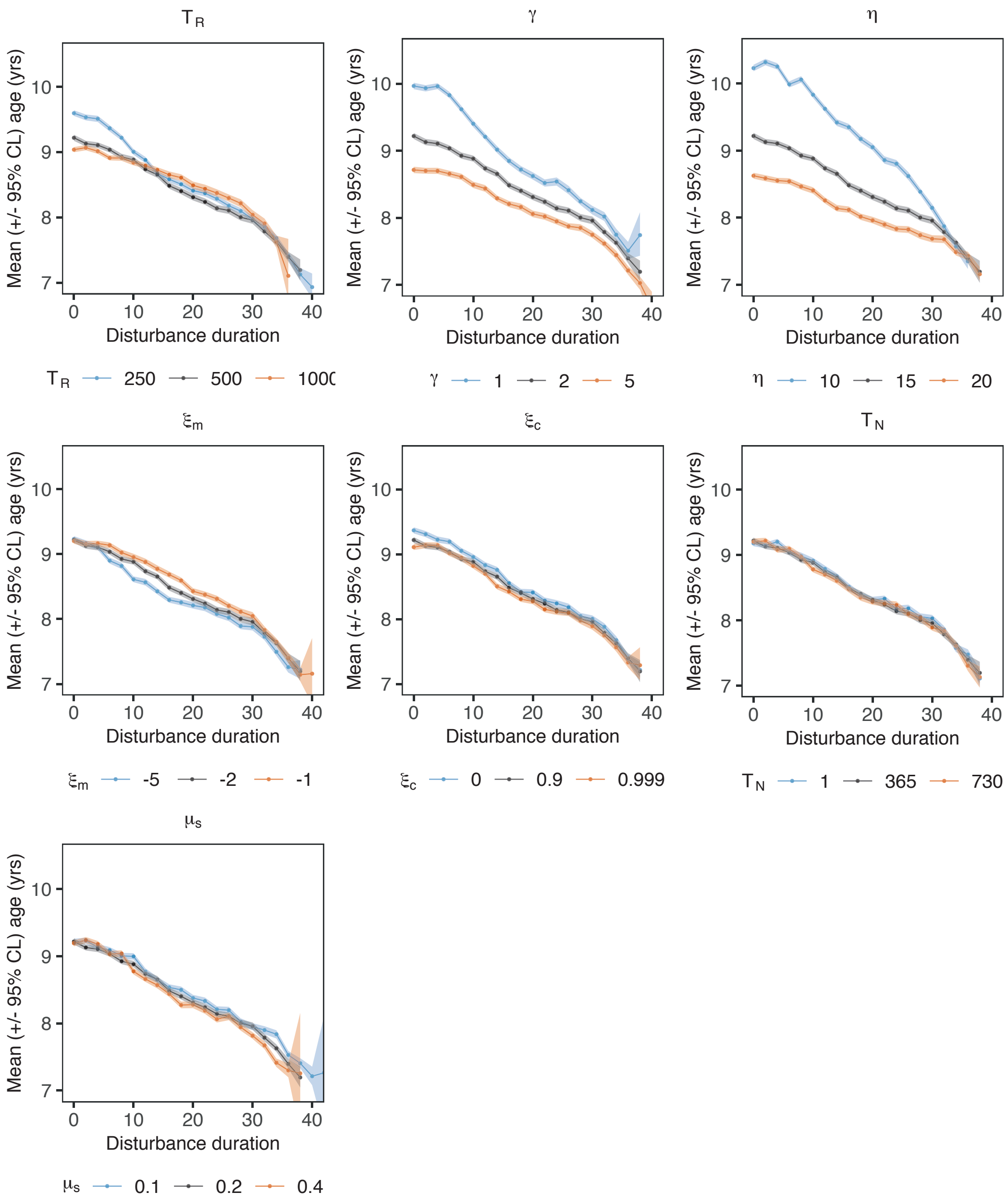

Figure 16: The effect of changes in $\eta, \gamma, T_R, \xi_m, \xi_c, T_N$ and μ_s on the response of the mean age at first reproduction to increasing disturbance duration. Points and lines indicate mean values, shading are the 95% confidence levels of the mean derived from nonparametric bootstrapping.

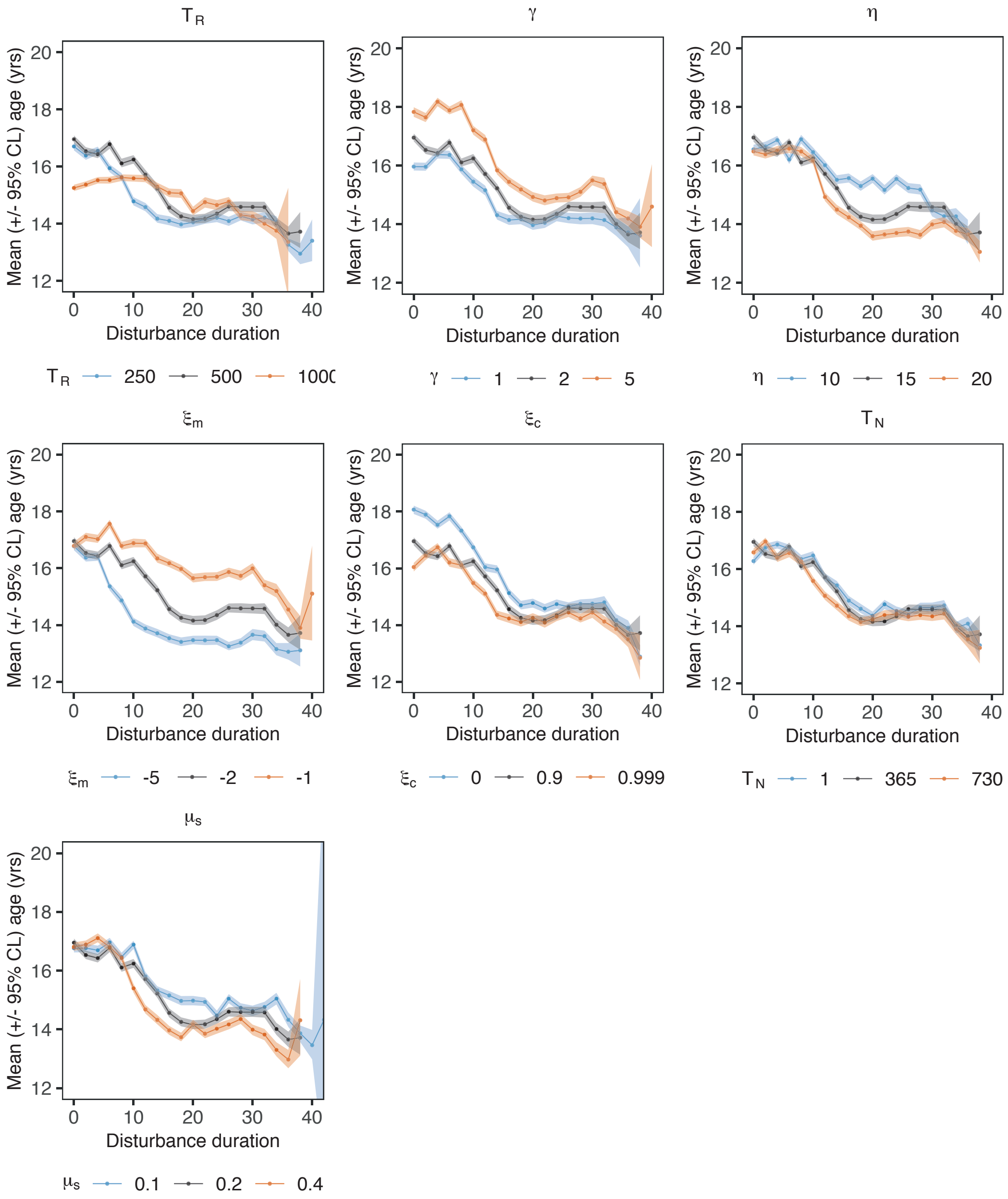

Figure 17: The effect of changes in $\eta, \gamma, T_R, \xi_m, \xi_c, T_N$ and μ_s on the response of the mean female age at weaning first calf to increasing disturbance duration. Points and lines as in Fig. 16.

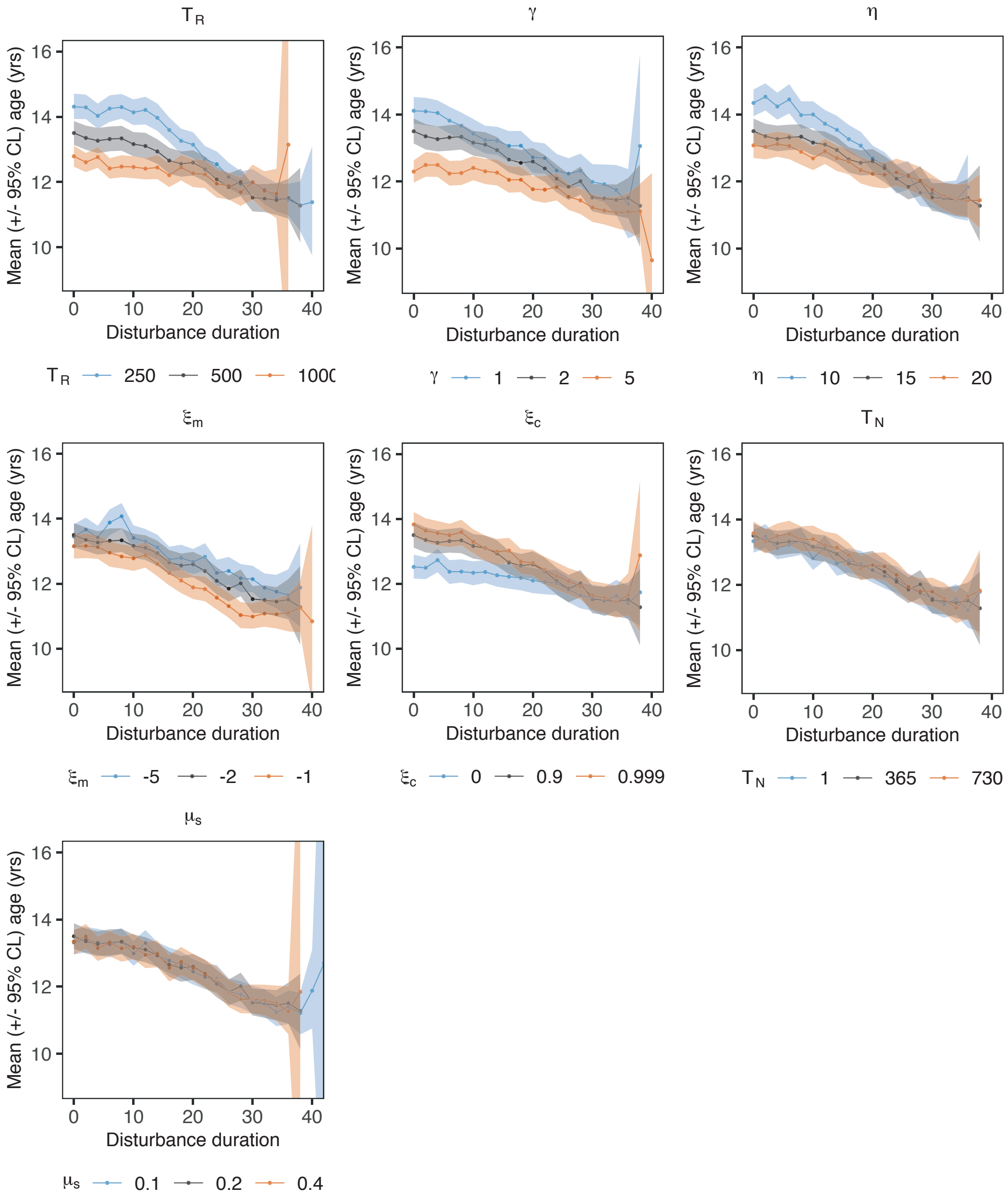

Figure 18: The effect of changes in $\eta, \gamma, T_R, \xi_m, \xi_c, T_N$ and μ_s on the response of life expectancy (mean female age at death) to increasing disturbance duration. Points and lines as in Fig. 16.

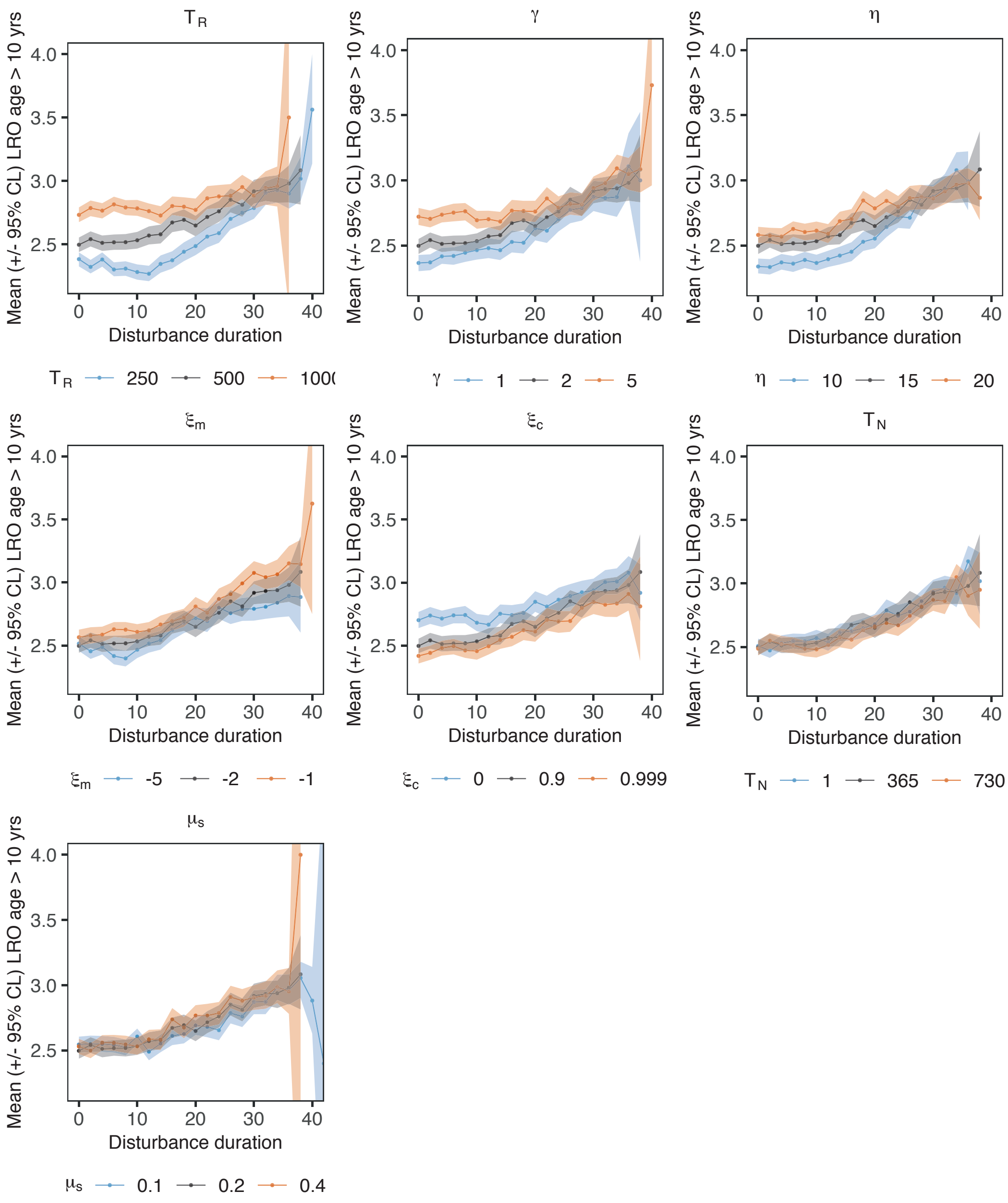

Figure 19: The effect of changes in $\eta, \gamma, T_R, \xi_m, \xi_c, T_N$ and μ_s on the response of the mean lifetime reproductive output of females that die beyond 10 yrs of age to increasing disturbance duration. Points and lines as in Fig. 16.
